# *Rhodococcus* strains as a good biotool for neutralizing pharmaceutical pollutants and obtaining therapeutically valuable products: Through the past into the future

**DOI:** 10.3389/fmicb.2022.967127

**Published:** 2022-09-29

**Authors:** Irina Ivshina, Grigory Bazhutin, Elena Tyumina

**Affiliations:** Perm Federal Research Center of the Ural Branch of the Russian Academy of Sciences, Perm, Russia

**Keywords:** Actinomycetes, *Rhodococcus*, pharmaceutical pollutants, biotransformation, biodegradation, therapeutically valuable products

## Abstract

Active pharmaceutical ingredients present a substantial risk when they reach the environment and drinking water sources. As a new type of dangerous pollutants with high chemical resistance and pronounced biological effects, they accumulate everywhere, often in significant concentrations (μg/L) in ecological environments, food chains, organs of farm animals and humans, and cause an intense response from the aquatic and soil microbiota. *Rhodococcus* spp. (*Actinomycetia* class), which occupy a dominant position in polluted ecosystems, stand out among other microorganisms with the greatest variety of degradable pollutants and participate in natural attenuation, are considered as active agents with high transforming and degrading impacts on pharmaceutical compounds. Many representatives of rhodococci are promising as unique sources of specific transforming enzymes, quorum quenching tools, natural products and novel antimicrobials, biosurfactants and nanostructures. The review presents the latest knowledge and current trends regarding the use of *Rhodococcus* spp. in the processes of pharmaceutical pollutants’ biodegradation, as well as in the fields of biocatalysis and biotechnology for the production of targeted pharmaceutical products. The current literature sources presented in the review can be helpful in future research programs aimed at promoting *Rhodococcus* spp. as potential biodegraders and biotransformers to control pharmaceutical pollution in the environment.

## Introduction

Active pharmaceutical ingredients are recognized contaminants of emerging concern and are ubiquitously found in aquatic and terrestrial environments. These molecules, not naturally occurring chemicals with highly stable chemical structures and pronounced biological activities, belong to a group of potentially dangerous mutagens. The problem of pharmaceutical pollution is becoming of a comprehensive planetary nature, posing a threat to the environment and human health (aus der Beek et al., 2016; Domingo-Echaburu et al., 2021; Wilkinson et al., 2022).

Pharmaceutical pollution occurs throughout the entire life cycle of an individual pharmaceutical compound, from its development and synthesis to consumption and disposal. It is known that, among other chemical industries, the pharmaceutical industry has the highest Environmental Impact Factor, reflecting the environmental impact of manufacturing processes (Sun et al., 2018). According to Kümmerer (2010), the synthesis of 1 kg of an active pharmaceutical ingredient generates 50–100 kg of waste. The control of drug pollution should be carried out at all stages, namely research & development, consumption, and disposal of pharmaceuticals. Modern drug discovery and development should not only exclude aggressive conditions and reagents and provide a minimum yield of by-products at each stage, but also predict the possible environmental fate of the drug being developed and its metabolites. This is the pivotal thesis of green and sustainable pharmacy (Kümmerer, 2010; Daughton and Ruhoy, 2014; Leder et al., 2015).

Along with the pharmaceutical industry, direct consumers of medicinal drugs are the source of environmental pollution by pharmaceutical residues. A real “drug boom,” widespread chronic illnesses, population aging, and prescription of drugs for preventative purposes, all contribute to an extraordinary rise in the human consumption of pharmaceuticals. According to the statistics obtained, half of all manufactured medicines are prescribed, distributed, sold, and improperly disposed of (Thomas and World Health Organization, 2017). Active pharmaceutical ingredients released into the environment have negative effects on invertebrates and vertebrates, microbiota and plants; they bioaccumulate within the food chain, disturb the structure and functioning of ecosystems (Oaks et al., 2004; Grenni et al., 2018; Mezzelani et al., 2018; Richmond et al., 2018; Giménez and Nunes, 2019; Aguilar-Romero et al., 2020; Drzymała and Kalka, 2020; Duarte et al., 2020; Grabarczyk et al., 2020; Wijaya et al., 2020; Markovic et al., 2021; Mulkiewicz et al., 2021; Rohonczy et al., 2021; You et al., 2021; Daniel et al., 2022).

These global environmental challenges make the transition to a bioeconomy—a biotechnology-based economy relying on renewable biological raw materials—inevitable (Marvik and Philp, 2020). The search for sustainable and environmentally friendly biomanufacturing has led to a surge in enzyme technologies, biocatalysis, and biodegradation approaches. Effective biocatalytic systems based on bacteria for obtaining valuable materials and pharmaceutical products are being actively developed as well as innovative biotechnology solutions for wastewater treatment and bioremediation of polluted territories are being implemented. The use of the enzymatic potential of microorganisms can significantly reduce energy consumption and environmental burden (Anteneh and Franco, 2019). It is also important that biological methods in addressing the problem of pharmaceutical pollution can be applied at opposite poles—both in the process of synthesis of pharmaceutically active compounds and in the process of degradation of pharmaceutical products.

Among the microorganisms capable of sensitive responses to adverse changes in their habitats, a special place is occupied by members of the actinomycete line of prokaryotic evolution—polyextremotolerant bacteria of the genus *Rhodococcus* Zopf 1891 (Approved Lists 1980; domain “*Bacteria*,” phylum *Actinomycetota*, class *Actinomycetia*, order *Mycobacteriales,* family *Nocardiaceae*; https://lpsn.dsmz.de/genus/rhodococcus).

Ecological geography and the range of possible habitats for rhodococci are unprecedentedly diverse. They are widely distributed in aquatic and terrestrial ecosystems across the world, including the Arctic and Antarctic (Zhang et al., 2015; Goordial et al., 2016). Rhodococci are isolated from clean and polluted soils, fresh surface water, groundwater and stratal water, domestic and industrial wastewater, mineral water, marine bottom sediments, rhizosphere and phyllosphere of plants, vertebrates and invertebrates, snow, air, and core samples (Bej et al., 2000; Ruberto et al., 2005; Li et al., 2012; Paterson et al., 2019; Sangal et al., 2019; Ivshina et al., 2021a; Martinez Alvarez et al., 2022). Most members of the genus *Rhodococcus* are free-living saprotrophs. Only two species have been identified as opportunistic pathogens—an animal and human pathogen *R*. *equi* (“*R*. *hoagie*”) and a phytopathogen *R*. *fascians*.

The widespread distribution of *Rhodococcus* spp. is ensured by their high tolerance to environmental stresses such as extreme temperatures, salinization, high concentrations of toxic metal(loid)s, desiccation, high/low pH, reactive oxygen species, and radiation exposure (Cappelletti et al., 2016; Presentato et al., 2018a; Firrincieli et al., 2019; Pátek et al., 2021). Understanding the complex mechanisms protecting rhodococci from environmental stresses as well as more detailed interpretation of the results of various stress responses that provide *Rhodococcus* resistance against many chemical compounds may open up new perspectives and result in a wide variety of their biotechnological applications in the near future.

*Rhodococcus* spp. are capable of oxidation and complete degradation of a wide range of organic compounds, recalcitrant pollutants and toxic compounds, pesticides, emerging pollutants, endocrine disruptors, explosives, flame retardants, plasticizers, defoliants, and plastics, including microplastics. Additionally, they have potential for fuel biodesulfurization, lipid synthesis, and biofuel production (Coleman et al., 2002; Gilan and Sivan, 2013; Auta et al., 2018; Presentato et al., 2018b; Pang et al., 2020; Alvarez et al., 2021; Hirschler et al., 2021; Keasling et al., 2021; Yu et al., 2021; Baek et al., 2022; Kamaraj et al., 2022). The biochemical potential of rhodococci is intensively studied due to their catabolic flexibility, unique enzymatic capabilities, broad substrate specificity and an effective set of survival strategies (Larkin et al., 2010; Cappelletti et al., 2019; de Carvalho, 2019; Kuyukina and Ivshina, 2019a). Features of rhodococci such as ecological generalization, autoaggregation, morphological differentiation, and altruistic cell death (when exposed to various stresses, the bacterial community might cause certain members of the population to die in order to favor the survival of the colony) provide their successful competitiveness and high survival in disturbed ecosystems (Hamamura et al., 2006; Ivshina et al., 2021a).

The genomic potential of actinomycetes, including rhodococci, is vastly superior to well-known chemical space. In other words, actinomycetes are capable of producing many more biologically valuable molecules than have been discovered so far (van Bergeijk et al., 2020). Rhodococci are characterized by a large genome (up to 12.7 Mb, which is more typical for *R*. *opacus* and *R*. *wratislaviensis*) containing information about numerous catabolic pathways for various chemical compounds; a significant degree of gene redundancy, which ensures functional stability of the genome; ring and linear plasmids, which represent an additional pool of DNA that can evolve and be easily transferred (Larkin et al., 2006, 2010; Haußmann et al., 2013; di Canito et al., 2018; Cappelletti et al., 2019). The above properties raise rhodococci to the rank of unique and promising biotechnology tools.

Several reviews have partially emphasized the biosynthetic capacities and potential for *Rhodococcus*-based biocatalysts to produce pharmaceutically valuable compounds (Anteneh and Franco, 2019; Busch et al., 2019; Cappelletti et al., 2020). Here, for the first time, we focus on rhodococci as potent agents in addressing the issue of pharmaceutical pollution, which is acute both during the development and production of an active pharmaceutical ingredient and during medicine consumption and pharma waste disposal. The purpose of this work was to provide a brief review and critical analysis of the recent data on the production of promising pharmaceutical products and the neutralization of pharmaceutical micro-pollutants using *Rhodococcus*-based biocatalysts.

## Biocatalysis of active pharmaceutical ingredients

Biocatalysis is a process by which enzymes or whole-cell systems carry out chemical transformations of organic molecules. Biocatalytic processes are effective, performed in mild conditions without forming toxic products, and, in some cases, cannot be replaced by traditional catalytic processes. Isolated and immobilized enzymes, cell lysates, and whole-cell systems can be used as biocatalysts (Woodley, 2008; Jiang and Fang, 2020). Biocatalysis is an attractive technology for the pharmaceutical industry. Biocatalysts provide excellent regio-, chemo-, stereo-, and enantioselectivity as well as improved product separation, lower costs, fewer side effects, less use of protective groups, and a better environmental impact(Lin and Tao, 2017; Devine et al., 2018; Sun et al., 2018). In addition, enzyme and whole-cell biocatalysts allow for challenging reactions. For example, biocatalysts can perform selective hydroxylation of inactivated carbon atoms—an extremely difficult challenge in terms of traditional chemistry (Narayan et al., 2015; de Carvalho, 2017; Dong et al., 2018).

Rhodococci are actively used in biocatalysis of pharmaceutical precursors and in novel drug development. Versatile *Rhodococcus* biocatalysts provide a wide range of chemical-enzymatic reactions while being an environmentally friendly approach (Anteneh and Franco, 2019; Busch et al., 2019; Cappelletti et al., 2020). Below, we consider specific examples of biocatalysis of pharmaceutically significant compounds using whole cells, individual enzymes, and heterologous expression systems.

### Whole-cell biocatalysts in organic biotransformations

Whole-cell biocatalysts provide several advantages. Firstly, the spectrum of compounds metabolized by whole cells is much wider than that by isolated enzymes. Therefore, using whole-cell biocatalysts, it is possible to synthesize a wide range of drugs from different therapeutic groups. Secondly, synthesis of valuable products by whole cells requires fewer steps (Tao and Xu, 2009; Sheldon and Woodley, 2018). Thirdly, whole-cell biocatalysts make it easy to implement enzymatic cascades spanning multiple reactions (Lin and Tao, 2017). Fourthly, whole-cell catalysts are less expensive than purified enzymes (Sakkos et al., 2019).

**Figure 1:**
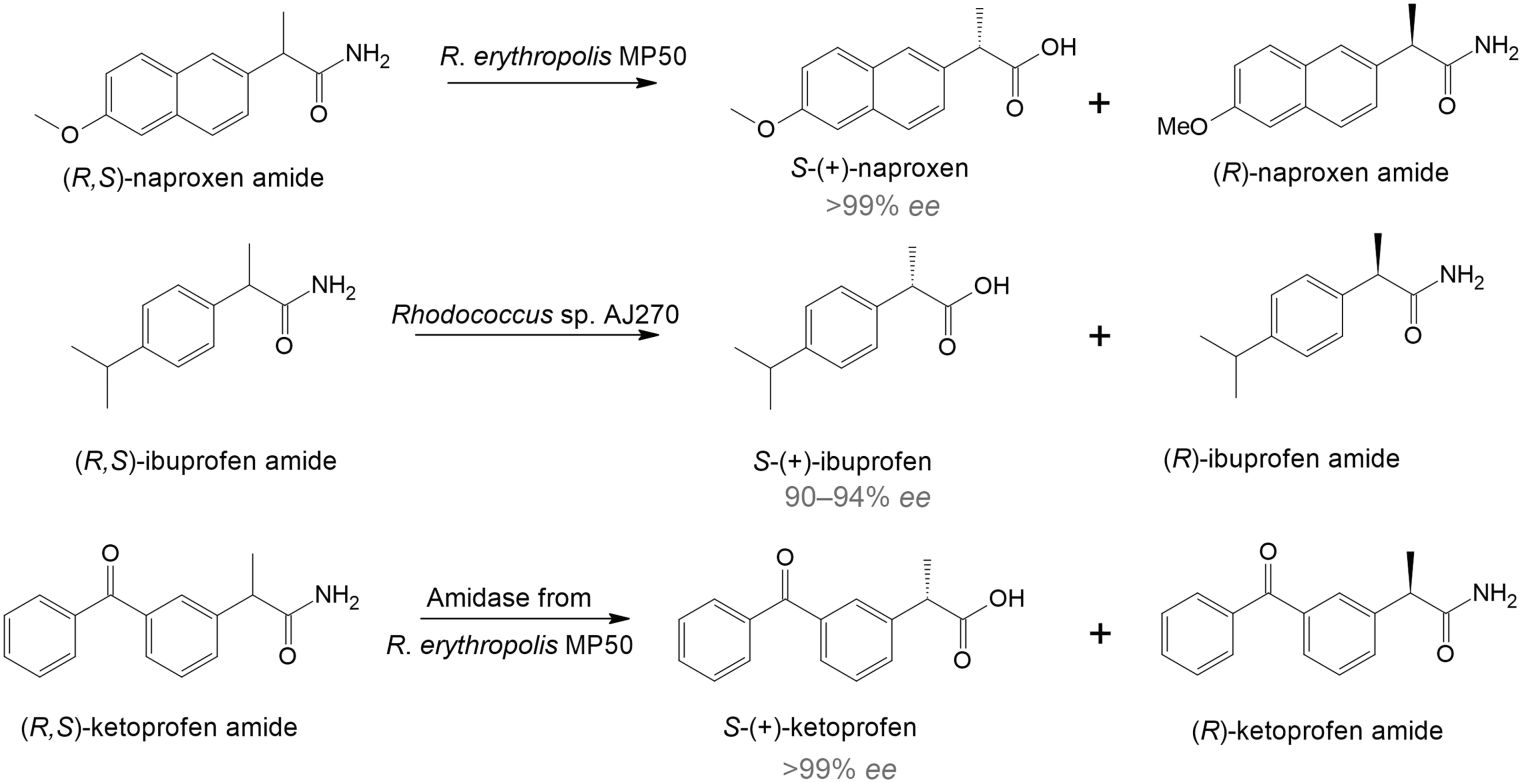
Enantiomeric hydrolysis of racemic amides to nonsteroidal anti-inflammatory drugs by rhodococci (Hirrlinger et al., 1996; Effenberger et al., 1997; Snell and Colby, 1999).

The use of rhodococci in bio-based techniques originated in the late 1990s and early 2000s and was associated with the synthesis of complex single-stereoisomer drugs (Devine et al., 2018). Most chiral drugs are racemic mixtures, in which one enantiomer is usually therapeutically active while the other is toxic. Therefore, it is crucial to obtain substances with a predominant active enantiomer (Anteneh and Franco, 2019). One of the effective ways to directly obtain enantiomeric pharmaceuticals is to use specific enzymatic systems as biocatalysts of regio- and stereoselective transformations. For example, racemic naproxen amide was hydrolyzed by immobilized *R*. *erythropolis* MP 50 cells with the formation of *S*-naproxen, a nonsteroidal anti-inflammatory drug that has a more pronounced therapeutic effect compared to *R*-naproxen (Effenberger et al., 1997; Figure 1). Another study describes the deracemization of ibuprofen. *Rhodococcus* sp. strain AJ270 transformed ibuprofen amide and four related 2-phenylpropionamides into *S*-(+)-ibuprofen with a high enantiomeric excess (90–94% *ee*; Snell and Colby, 1999). Moreover, the authors found that the main enzymes involved in this reaction were amidases.

**Figure 2:**
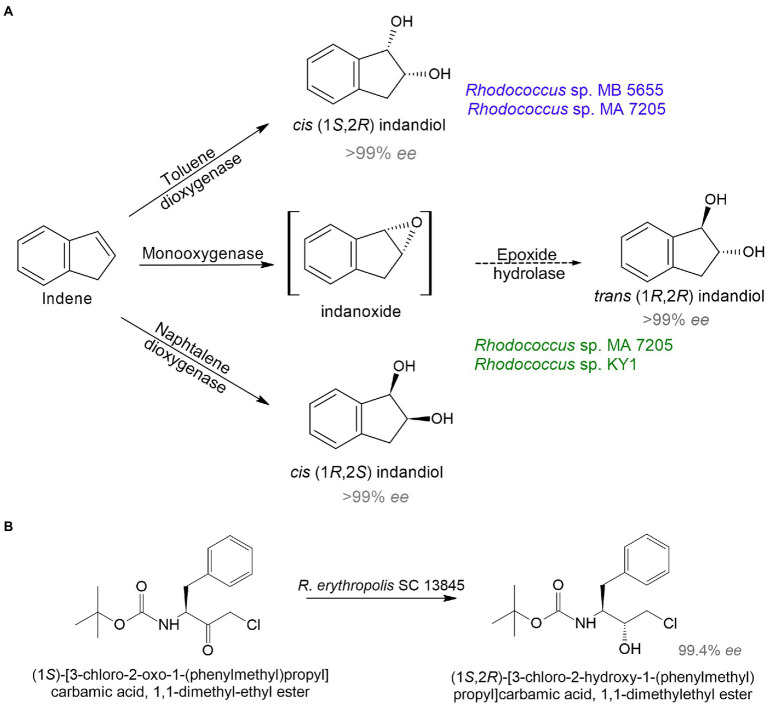
Bioproduction of precursors of HIV medicines indinavir **(A)** and atazanavir **(B)** by rhodococci (Chartrain et al., 1998; Stafford et al., 2001; Patel et al., 2003).

Because of the need for therapeutics to treat conditions such as infectious diseases, diabetes, cancer, and mental disorders, the development of new highly effective drugs and their precursors is of particular interest. *Rhodococcus* sp. strain B 264-1 (MB 5655) and strain I-24 (MA 7205) transformed indene into *cis*-(1*S*,2*R*)-indandiol and *trans*-(1*R*,2*R*)-indandiol with 98–99% *ee* (Chartrain et al., 1998; Figure 2A). Indanediol is a precursor of indinavir—a medicine prescribed for HIV treatment. Further, continuous flow cultivation of the strain I-24 resulted in the evolution of a mutant *Rhodococcus* sp. KY1 with better bioconversion capabilities, including a twofold increase in *trans*(1*R*,2*R*)-indandiol production (Stafford et al., 2001). The key intermediate for synthesis of atazanavir—another HIV medication—*tert*-butyl ((2*S*,3*R*)-4-chloro-3-hydroxy-1-phenylbutane-2-yl)carbamate was obtained using *R. erythropolis* SC 13854 with 95% yield and 99% *ee* (Patel et al., 2003; Figure 2B).

**Figure 3:**
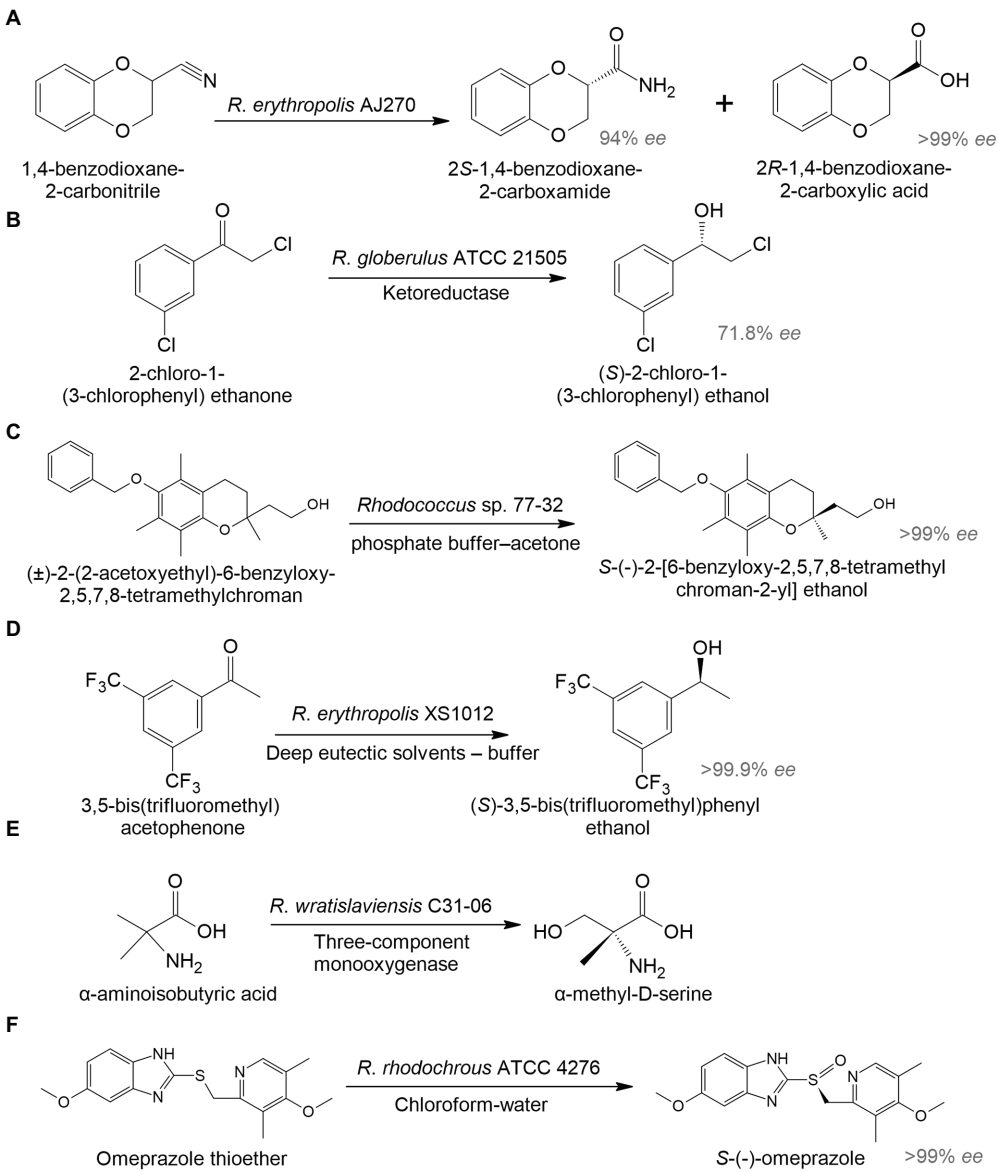
Biocatalysis of 2R-1,4-benzodioxane-2-carboxylic acid (A), (S)-2-chloro-1-(3-chlorophenyl) ethanol (B), S-(-)-2-[6-benzyloxy-2,5,7,8-tetramethylchroman-2-yl] ethanol (C), (S)-3,5-bis(trifluoromethyl)phenyl ethanol (D), α-methyl-D-serine (E), and esomeprazole (F) by whole-cell Rhodococcus spp. (Hanson et al., 2005; Liu et al., 2006; Petukhova et al., 2017; Chen et al., 2020; Hibi et al., 2021; Zhang et al., 2021b).

In addition to antiretroviral medicines, there were originally studies on the biosynthesis of drugs for the treatment of cancer and cardiovascular diseases. An interesting example illustrated the hydrolysis of (*R*)1,4-dibenzodioxane-2-carbonitrile to 2*S*-1,4-benzodioxane-2-carboxamide and 2*R*-1,4-benzodioxane-2-carboxylic acid by *R*. *erythropolis* AJ270 (Liu et al., 2006). 2*R*-1,4-benzodioxane-2-carboxylic acid is used to produce a hypotensive and vasodilating drug doxazosin (Figure 3A). In another study, *R. globerulus* SC 16305 produced (*S*)-2-chloro-1-(3-chlorophenyl)ethanol (71.8% *ee*), which is used in the synthesis of an IGF-1 receptor inhibitor as an anticancer therapy (Hanson et al., 2005; Figure 3B).

There has been increasing interest in using biocatalysis to develop drugs for the treatment of mental illnesses. The enantiomeric transformation of *S*-(-)-2-[6-benzyloxy-2,5,7,8-tetramethylchroman-2-yl] ethanol from a racemic mixture was achieved using a whole-cell biocatalyst based on *Rhodococcus* sp. 77-32 (Figure 3C; Petukhova et al., 2017). The resulting enantiomer can be used in the preparation of α-tocols exhibiting cytoprotective and antioxidant properties and reducing neuroinflammation in the brain (Muscaritoli, 2021). According to Chen et al. (2020), *R. erythropolis* XS1012 transformed 3,5-bis(trifluoromethyl) acetophenone into (*S*)-3,5-bistrifluoromethylphenyl ethanol, which is a key pharmaceutical intermediate of the neurokinin-1 receptor antagonist with antidepressant and anxiolytic properties (Figure 3D). In another study, *R. wratislaviensis* C31-06 transformed α-aminoisobutyric acid into α-methyl-D-serine (Hibi et al., 2021; Figure 3E). The latter may be a potential therapeutic agent and/or biomarker for some mental disorders (MacKay et al., 2019). Proteomic analysis revealed the involvement of a three-component monooxygenase consisting of an amido hydroxylase component (AibH1H2) and an electron transfer component (AibF and AibG).

To enhance the target product synthesis in complex multi-stage biochemical transformations of the initial substrates, immobilized biocatalysts are used. *Rhodococcus*-based biocatalysts immobilized on various carriers can be repeatedly applied across a wide range of temperatures, pH values, and pressures. For example, alginate-immobilized *R. rhodochrous* ATCC 4276 cells in a biphasic system (chloroform-water) transformed omeprazole thioether into esomeprazole with 94.8% yield and > 99% *ee*. It is a popular proton-pump inhibitor used in the treatment of gastrointestinal ulcers (Zhang et al., 2021b; Figure 3F).

**Figure 4:**
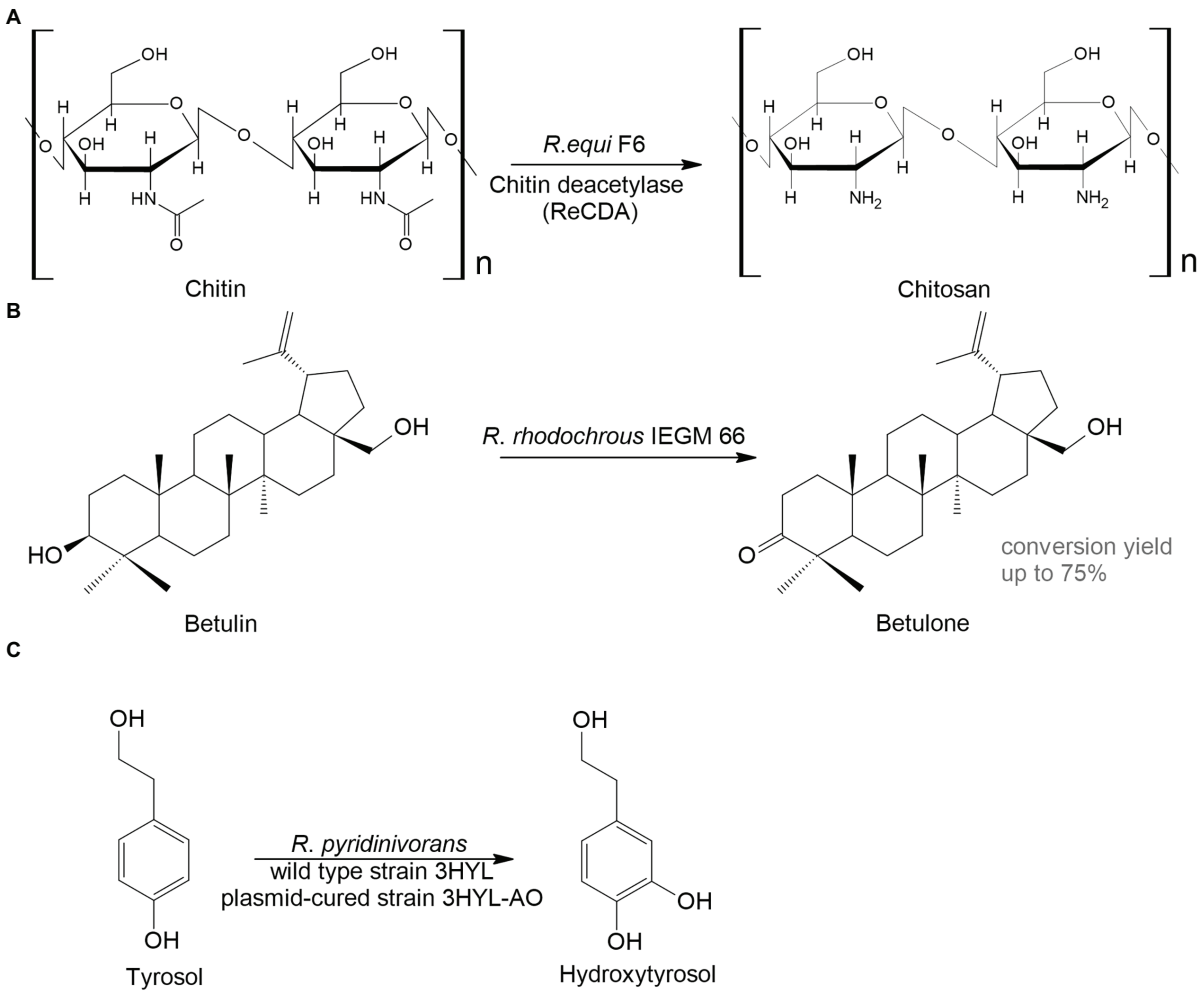
*Rhodococcus* bioconversion of animal – **(A)** and plant-derived **(B,C)** compounds (Grishko et al., 2013; Ma et al., 2020; Anissi et al., 2021).

Another interesting area for biocatalysts is the conversion of animal- and plant-derived medicines. Chitosan is a linear polysaccharide that has antitumor, immunoenhancing, antifungal, antimicrobial, antioxidant, and wound healing activities and is widely used in biomedicine and the pharmaceutical industry (Shariatinia, 2019). The basic method to obtain chitosan is by treating crustacean-derived chitin with thermo-alkaline, which is environmentally unsafe. An alternative option is the enzymatic transformation of chitin. For example, Ma et al. (2020) isolated *Rhodococcus equi* F6, which transformed chitin into chitosan. The main transformation enzyme was chitin deacetylase (Figure 4A).

Plant metabolites are abundant sources of bioactive compounds that have a variety of positive impacts on human health. However, the extraction and isolation of these chemicals are laborious, time-consuming and energy-intensive. A great alternative to this is microbial biocatalysis. Here are two examples to illustrate this. Betulone is an oxo-derived betulin—plant pentacyclic triterpenoid—that exhibits antitumor, anti-inflammatory, antiparasitic, anticancer, and anti-HIV properties (Bębenek et al., 2018; Kolobova et al., 2019; Darme et al., 2022). The screening of collection strains showed that growing and resting *R. rhodochrous* IEGM 66 cells were capable of efficient transformation of up to 3 g/L of the triterpenoid betulin into betulone with a yield of 40–75% (Grishko et al., 2013; Figure 4B).

Hydroxytyrosol, *o*-diphenol, found in olive trees, has antithrombotic, antioxidant, antiviral, and anticancer activities. Extraction of the compound from plants is difficult; therefore, it is advantageous to use bacterial biocatalysts. The wild type or the plasmid-harboring *R. pyridinivorans* strain 3HYL transformed tyrosol into hydroxytyrosol at concentrations of 16.4 ± 0.23 mmol/L and 23.01 ± 2.37 mmol/L, respectively (Anissi et al., 2021; Figure 4C).

Although relatively simple and frequently used, whole-cell processes have a significant disadvantage since the target catalytic reaction is influenced by other metabolic reactions, leading to other by-products that affect the separation and purification of target products (Şahin, 2019; Han et al., 2021). This can be avoided by using isolated enzymes.

### Enzyme biocatalysts for chemical transformations of organic compounds

In biotechnology, enzymes draw attention due to their high activity, non-toxicity, and biodegradability. Furthermore, since enzymatic biocatalysts are characterized by exceptional selectivity, substrate specificity, and activity under mild conditions, there is no need for protection and deprotection of functional groups and chiral centers in molecules (Woodley, 2008). Application of enzymatic biocatalysts is challenged because of a narrow range of metabolized substrates, limited stability under extreme environmental conditions, as well as low yields of target products (up to 50%) in one reaction cycle. However, the use of asymmetric enzymes may lead to a 100% yield.

Rhodococci are a source of a variety of economically important enzymes. Recently, Busch et al. (2019) conducted a comprehensive review of the most promising enzymes of rhodococci involved in fine organic synthesis. The information about *Rhodococcus*-derived enzymes (isolated and heterologously expressed) used for the synthesis of therapeutically valuable compounds is summarized in Table 1.

**Table 1:** Pharmaceutically important enzymes produced in *Rhodococcus* spp.

Enzyme	Catalyzed reaction	Source	Reference
*Oxidoreductases*			
Alcohol dehydrogenase	Interconversion between alcohols and aldehydes or ketones	*Rhodococcus* sp. R6 (RhADH) *R. erythropolis* DSM 43297 (ReADH) *R*. *ruber* (ADH-A) *R. kyotonensis* (Rhky-ADH) *R*. *erythropolis* MAK154	Yamamura (2018a), Dhoke et al. (2020), Hu et al. (2020), Han et al. (2021), Xu et al. (2022)
Carbonyl reductase	Reduction of aliphatic aldehydes and ketones	*R. pyridinivorans* (RpCR) *R*. *globerulus* ATCC 21505	Hanson et al. (2005), Xu et al. (2016)
Cholesterol oxidase	Oxidation of cholesterol to cholestenone	*R*. *erythropolis* CECT3014 (ChoG) *R*. *ruber* Chol-4 (ChoG)	Fernández de las Heras et al. (2011, 2014)
Hydroxysteroid dehydrogenase	Oxidoreduction of the hydroxyl/carbonyl groups on the core structure of hydroxysteroids	*R. ruber* (12α-HSDH)	Shi et al. (2019)
Ketosteroid dehydrogenase	Introduction of a double bond between the C1 and C2 atoms in the A ring of 3-ketosteroid substrates	*R*. *ruber* Chol-4	Fernández de las Heras et al. (2012)
Ketosteroid hydroxylase	Cleavage of the steroid polycyclic ring by C9a-hydroxylation	*R. erythropolis* SQ1 *R. ruber* Chol-4	van der Geize et al. (2002), Guevara et al. (2017)
Monooxygenase	Incorporation of one atom of the oxygen molecule into the substrate	*R. wratislaviensis* C31-06 (AibH1H2, AibG, and AibF)	Hibi et al. (2021)
Naphtalene dioxygenase	Conversion of naphnalene into cis-dihydrodiols	*Rhodococcus* sp. MA7205 *Rhodococcus* sp. KY1	Stafford et al. (2001)
Phenylalanine dehydrogenase	Oxidative deamination of L-phenylalanine to phenylpyruvate	*Rhodococcus* sp. M4 (pheDH)	Ye et al. (2015)
Putrescine oxidase	Oxidation of putrescine, other aliphatic diamines, amino alcohols and polyamines	*R. erythropolis* (Re-PuO)	Anyanwu et al. (2021)
Toluene dioxygenase	Conversion of toluenes into cis-dihydrodiols	*Rhodococcus* sp. MB 5655, MA7205	Chartrain et al. (1998)
*Hydrolases*			
Amidase	Conversion of amides, to ammonia and carboxylic acids	R*. erythropolis* MP50 *R. erythropolis* A4	Hirrlinger et al. (1996), Rucká et al. (2014)
Chitin deacetylase	Hydrolysis of the N-acetamido groups of N-acetyl-D-glucosamine residues in chitin	*Rhodococcus equi* F6 (ReCDA)	Ma et al. (2020)
Nitrilase	Direct conversion of nitriles into carboxylic acids and ammonia	*R. zopfii* (*Rz*NIT/W167G)	Dai et al. (2022a,b)
*Lyases*			
Aldoxime dehydratase	Dehydration of an aldoxime to a nitrile	*Rhodococcus* sp. YH3-3 (OxdYH3-3)	Choi et al. (2019)
Benzaldehyde lyase	Reversible cleavage of (*R*)-benzoin to benzaldehyde	*R. erythropolis* R138 (*Re*BAL)	Peng et al. (2021)
Nitrile hydratase	Formation of amides from nitriles	*R. erythropolis* A4	Rucká et al. (2014)

There are three main classes of enzymes involved in pharmacologically important reactions: oxidoreductases, hydrolases, and lyases. Among oxidoreductases, alcohol dehydrogenases are of special interest. They carry out a reversible reduction of ketones to alcohols. These enzymes mediate highly efficient syntheses of chiral aromatic alcohols, which are important building blocks in the pharmaceutical industry. For instance, alcohol dehydrogenase RhADH from *Rhodococcus* sp. R6 catalyzed the reaction of asymmetric reduction of aromatic ketones to chiral aromatic alcohols. Thus, 2-hydroxyacetophenone was successfully converted to (*R*)-(-)-1-phenyl-1,2-ethanediol (99% *ee*), an important pharmaceutical precursor (Han et al., 2021). The zinc-dependent alcohol dehydrogenase from *R. erythropolis* DSM 43297 (ReADH) converted ethyl 2-oxo-4-phenylbutyrate to ethyl 2-hydroxy-4-phenylbutanoate, an important intermediate for anti-hypertension drugs such as enalaprilat and lisinopril (Dhoke et al., 2020).

To improve the efficiency of biocatalytic reactions, it is advisable to use cascade enzyme systems. An enzyme cascade consists of several enzymes where the product of one enzyme serves as the substrate for the next. Thus, phenylethanols from ethylbenzene and its derivatives were synthesized using a bienzymatic cascade (Xu et al., 2022). Peroxygenase from the agaricomycete *Agrocybe aegerita* was used to oxidize ethylbenzene and its derivatives to ketones, which were further reduced to (*R*)- or (*S*)-phenylethanols using (*R*)-selective alcohol dehydrogenase from *Lactobacillus kefir* or (*S*)-selective alcohol dehydrogenase from *R. ruber*, respectively. Another example is the use of a bienzyme–coupled catalytic system composed of *Rhodococcus* sp. R6 alcohol dehydrogenase RhADH and *Candida parapsilosis* C5 formate dehydrogenase (Han et al., 2021). This enzyme complex catalyzed the asymmetric reduction of 2’-hydroxyacetophenone to (*R*)-(-)-1-phenyl-1,2-ethanediol with 99% *ee*.

Other important building blocks in the synthesis of pharmaceutical compounds are nitrogen-containing heterocycles (Naicker et al., 2015; Yang, 2015). Putrescine oxidase from *R. erythropolis* (Re-PuO) catalyzed the oxidation of diamines cadaverine and putrescine to N-heterocycle 1-piperideine, a building block in small-molecule active pharmaceuticals (Anyanwu et al., 2021).

### Heterologous transformation of organic compounds

**Figure 5:**
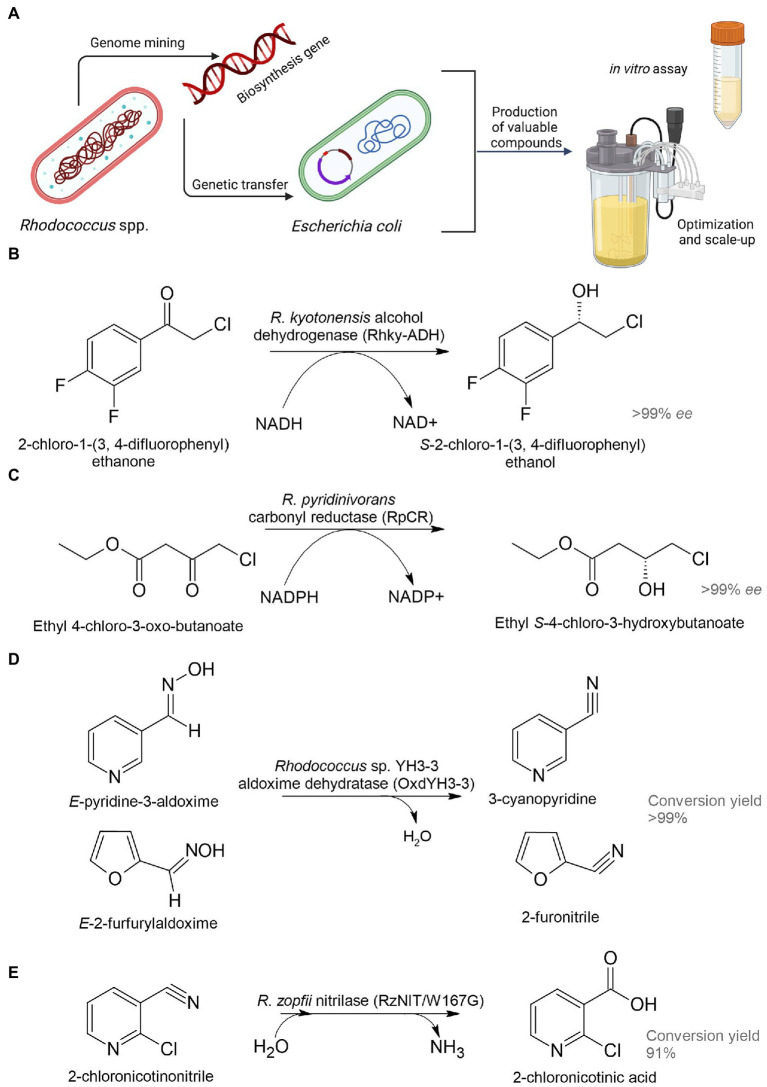
Heterologous biosynthesis of pharmaceutical precursors. Schematic representation of heterologous expression system **(A)**; biosynthesis of (*S*)-2-chloro-1-(3,4-difluorophenyl) ethanol **(B)**, ethyl (*S*)-4-chloro-3-hydroxybutanoate **(C)**, 3-cyanopyridine and 2-furonitrile **(D)**, and 2-chloronicotinic acid **(E)** (Xu et al., 2016; Choi et al., 2019; Hu et al., 2020; Dai et al., 2022b).

The rapid development of recombinant DNA technologies has led to the shift from native cells to recombinant cell factories that include heterologous enzymes and/or synthetic, orthologous pathways for the synthesis of important industrial compounds (Ladkau et al., 2014). Heterologous transformation is the engineering of conversion of organic chemicals in a technically convenient surrogate host (relative to the native producer) through the application of genetics and molecular biology (Figure 5A). *Escherichia coli* is the most widely used microbial platform for cell factories due to its well-studied genome, a powerful set of molecular biological tools for metabolic engineering and highly developed fermentation processes with inexpensive raw materials (Lin and Tao, 2017).

Dehydrogenase (named Rhky-ADH) was identified from *R. kyotonensis* and expressed in *E. coli* (Figure 5B). Using such a whole-cell biocatalyst, transformation of 2-chloro-1-(3,4-difluorophenyl) ethanone to (*S*)-2-chloro-1-(3,4-difluorophenyl) ethanol with more than 99% *ee* and 99% conversion was achieved (see Table 1). This compound is used for the synthesis of an antiplatelet agent ticagrelor (Hu et al., 2020). 12α-Hydroxysteroid dehydrogenase (12α-HSDH) from *R. ruber* expressed in *E. coli* had the potential to convert cheap and readily available cholic acid to 12-oxochenodeoxycholic acid (71–85% yields), which is a key precursor for chemoenzymatic synthesis of a therapeutic bile acid ursodeoxycholic acid (Shi et al., 2019). *Rhodococcus* phenylalanine dehydrogenase (pheDH) was engineered by directed evolution and expressed in *E. coli* for the highly enantioselective reductive amination of phenylacetone and 4-phenyl-2-butanone, giving (*R*)-amphetamine and (*R*)-1-methyl-3-phenylpropylamine in >98% *ee*, respectively (Ye et al., 2015). (*R*)-amphetamine is a precursor of a bronchodilator (*R*,*R*)-formoterol and a prostate drug tamsulosin; (*R*)-1-methyl-3-phenylpropylamine is a precursor of an antihypertensive dilevalol.

Analysis of the *R. pyridinivorans* genome identified an NADPH-dependent carbonyl reductase (RpCR) capable of asymmetric reduction of ethyl 4-chloro-3-oxobutanoate (Xu et al., 2016). RpCR was expressed in *E. coli* BL21(DE3) to produce ethyl (*S*)-4-chloro-3-hydroxybutanoate (>99% *ee*) without the addition of an external cofactor, resulting in a 91% molar isolation yield (Figure 5C). This compound is a universal and important chiral intermediate to produce chiral drugs, including cholesterol-lowering 3-hydroxy-3-methyl-glutaryl-CoA reductase inhibitors (statins).

The gene encoding aldoxime dehydratase from *Rhodococcus* sp. YH3-3 was expressed in *E. soli* in order to achieve an effective conversion of E-pyridine-3-aldoxime into the pharmaceutical precursor 3-cyanopyridine (Figure 5D; Choi et al., 2019). 2-Chloronicotinic acid is an important precursor for the production of the antidepressant mirtazapine and the antiviral agent nevirapine. A mutant of nitrilase (*Rz*NIT/W167G) from *R. zopfii* was expressed in *E. coli* for the heterologous synthesis of 2-chloronicotinic acid from 2-chloronicotinonitrile (Dai et al., 2022a,b). Accumulation of 2-chloronicotinic acid was 318.5 mM with a yield of 91% for 50 h, which is by far the highest level of biosynthesis of this compound catalyzed by nitrilase (Figure 5E). Rucká et al. (2014) investigated a cluster of genes encoding nitrile hydratase and amidase from *R. erythropolis* A4. These enzymes can be used for effective hydrolysis of cyanohydrins to form pharmaceutically important 2-hydroxy amides and 2-hydroxy acids.

A few studies reported on *Rhodococcus* spp. as hosts for the recombinant production of pharmaceutical precursors. For example, genes encoding carbonyl reductase from the methylotrophic yeast *Ogataea minuta* (OCR_V166A) and NADP^+^-dependent secondary alcohol dehydrogenase from the Gram-positive bacterium *Thermoanaerobacter ethanolicus* (TeADH) were co-expressed in *R. opacus* B-4, and the recombinant cells were used as lipophilic whole-cell biocatalysts to convert trifluoroacetophenone to α-(trifluoromethyl)benzyl alcohol (Honda et al., 2019). Yamamura (2019) designed a *Rhodococcus* expression system based on *R. erythropolis* JCM3191, which converted pyridoxine to pyridoxamine (a type of vitamin B_6_) through pyridoxal. For this purpose, genes encoding pyridoxine 4-oxidase and pyridoxamine-pyruvate aminotransferase from the proteobacterium *Mesorhizobium loti* were expressed in JCM391. Another study reported the efficient biocatalytic synthesis of 25-hydroxyvitamin D_3_ and 1α,25-dihydroxyvitamin, important hydroxylated derivatives to treat vitamin D_3_ deficiency (Yasutake et al., 2013). *R. erythropolis* JCM 3201 containing a mutant of vitamin D_3_ hydroxylase (Vdh _T107A_) from another actinomycete, *Pseudonocardia autotrophica*, was used as a biocatalyst.

To match biocatalytic technologies with modern scales and industry requirements, the development of effective toolkits for engineering *Rhodococcus* strainsis an urgent issue. Although rhodococci natively have powerful technological advantages, the productivity of wild-type strains under standard laboratory conditions leaves much to be desired. Factors such as high (61–71%) GC content, a thick cell wall and difficult-to-isolate genetic material, low transformation and recombination efficiencies prevent the production of stable and highly productive *Rhodococcus* biocatalysts. To overcome these obstacles, gene transfer techniques and genome editing tools (for instance, the CRISPR/Cas9 and CRISPRI systems) have been recently developed (DeLorenzo et al., 2021; Liang and Yu, 2021). Yamamura (2018a) performed genetic manipulations of *R. erythropolis* MAK154 for homologous expression (overexpression of autologous genes) of aminoalcohol dehydrogenase genes (*aadh*) that produce double chiral compounds—important building blocks for pharmaceuticals. He constructed *Rhodococcus* expression vectors derived from the *Rhodococcus*–*E. coli* shuttle vector pRET1102 and different promoters to increase the expression of *aadh*. This vector was successfully transformed into many actinomycete strains (Yamamura, 2018a,b).

Currently, studies are underway using machine learning to search for genes encoding pharmaceutically useful enzymes. Thus, for example, the computational prediction tool Tome enabled the identification of a thermostable benzaldehyde lyase (*Re*BAL) from *R. erythropolis* R138 (Peng et al., 2021). To expand the substrate spectrum of the enzyme, the authors used site-directed mutagenesis in the binding site of the gene encoding for *Re*BAL to form two variants of *Re*BAL_mat_ and *Re*BAL_wid_.

## Biocatalysis of steroids

Steroids are a group of terpenoid lipids widely distributed in nature (Fernández-Cabezón et al., 2018; Guevara et al., 2019b). They are perhydro-1,2-cyclopentanophenanthrene derivatives and include sterols, bile acids, steroid hormones, cardenolides, sapogenins, saponins, and vitamin D derivatives (Olivera and Luengo, 2019). Steroids are active ingredients of many medications, including anti-inflammatory, immunosuppressive, anti-allergic, anti-cancer, diuretic, anabolic, contraceptive, anti-androgenic, and pregestational agents (Costa et al., 2020; Felpeto-Santero et al., 2021). Traditional methods of obtaining steroids by extraction from plants and animals or organic synthesis are inefficient, multi-stage, labor-consuming, and environmentally unsafe (Olivera and Luengo, 2019). Microbiological transformation of steroids from low-cost precursors with the formation of key pharmaceutical intermediates, such as androst-4-en-3,17-dione (AD) and androst-1,4-diene-3,17-dione (ADD), is a successful biocatalytic technology used in the pharmaceutical industry.

Along with metabolites formed during steroid bioconversion, novel catalytic properties of microorganisms towards steroids are constantly being found. Rhodococci are able to carry out different reactions of steroid modifications: side-chain degradation, hydroxylations (e.g., at positions 9α, 16α, 7α, and 11β), insertion of C-C-double bonds (e.g., ∆1 and ∆4), hydrogenation and isomerization of double bonds (e.g., ∆5 → ∆4), carbonyl group oxidation/reduction (e.g., at positions 17 and 20), hydrolysis of steroid esters (e.g., 21-deacetylation; Donova, 2017). This is possible, firstly, due to the thick lipophilic cell wall of rhodococci, which facilitates hydrophobic steroid compounds to easily penetrate into the cell for further enzymatic transformation, and secondly, due to the presence of special transformation and degradation enzymes that carry out a sequential cascade of reactions.

**Figure 6:**
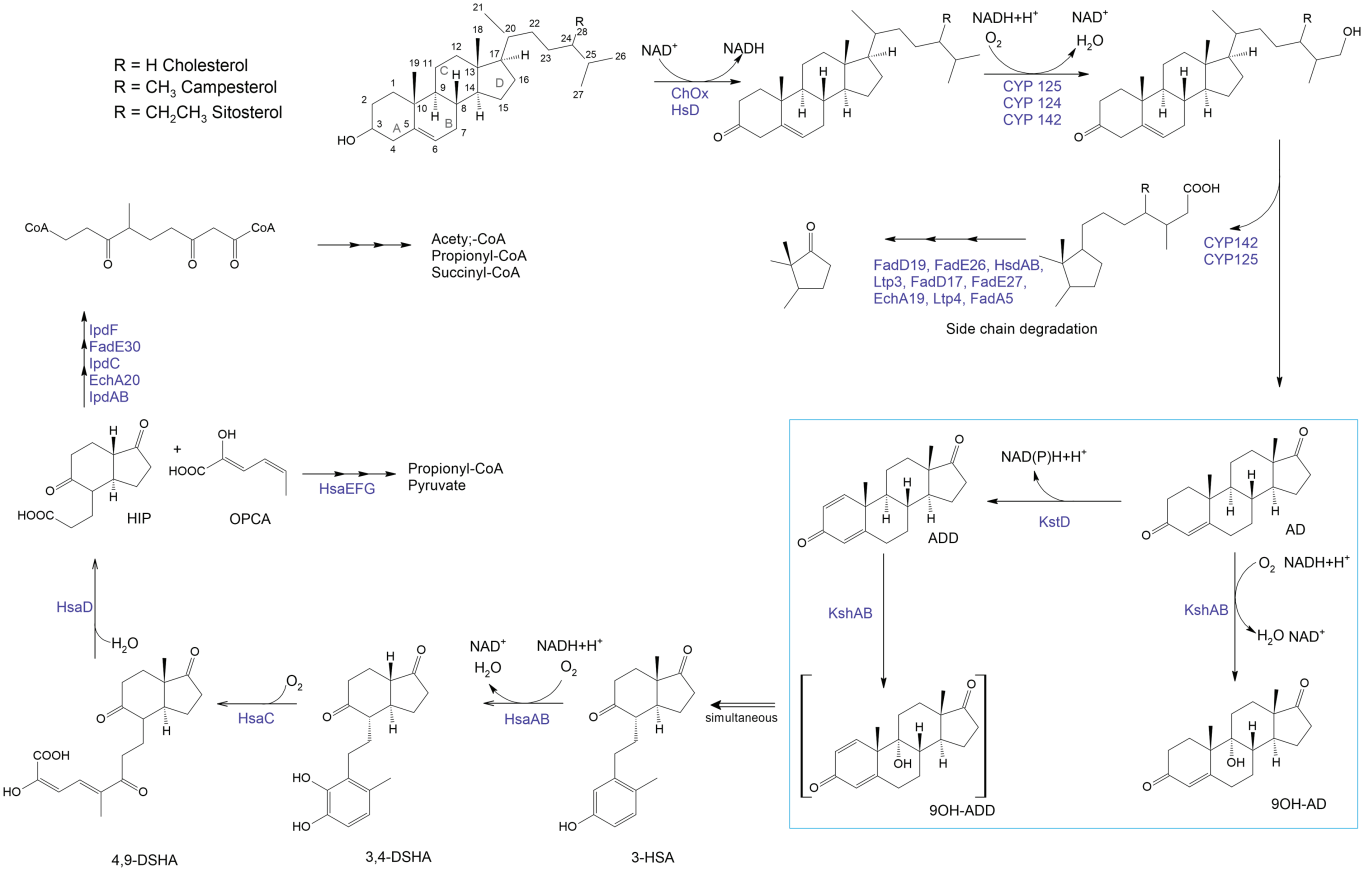
Aerobic sterol degradation in rhodococci and other actinomycetes. It starts with sterol being converted to stenone, which is catalyzed by a cholesterol oxidase (ChOx) or a hydroxysteroid dehydrogenase (HsD). Further, side chain degradation occurs with ω-methyl oxidation by cytochrome P450 monooxygenases (CYP 142, CYP 125) and β-oxidation-like reactions which lead to the formation of propionyl-CoA and acetyl-CoA (central metabolites). The steroid nucleus degradation involves oxygen-dependent rupture and subsequent hydrolytic cleavage of rings A and B following the 9,10-seco pathway due to 3-ketosteroid-dehydrogenase (KstD) and 3-ketosteroid 9α-hydroxylase (KsH). AD – 4-androstene-3,17-dione; ADD – androsta-1,4-diene-3,17-dione; 9OH-AD –9α-hydroxy-4-androstene-3,17-dione; 9OH-ADD – 9 α -hydroxyandrosta-1,4-diene-3,17-dione; 3-HSA – 3-hydroxy-9,10-secoandrosta-1,3,5(10)-triene-9,17-dione; 3,4-DHSA – 3,4-dihydroxy-9,10-secoandrosta-1,3,5(10)-triene-9,17-dione; 4,9-DSHA – 4,5,9,10-diseco-3-hydroxy-5-9-17-trioxoandrosta-1(10),2-diene-4-oic acid; HIP – 3aα-H-4α (3′-propanoate)-7a-β-methylhexahydro-1,5-indanedione; OPCA – oxypentadienecarboxylic acid (Haußmann et al., 2013; Kreit, 2017; Baldanta et al., 2021).

Biocatalytic transformations begin with the oxidation of steroids to stenones by a cholesterol oxidase (ChOx; Baldanta et al., 2021; Figure 6). ChOx is an extracellular flavin-dependent bifunctional oxidoreductase catalyzing the oxidation of a Δ^5^-ene-3β-hydroxysterol to Δ^5^-ene-3β-ketosteroid and the isomerization of the latter to yield Δ^4^-ene-3β-ketosteroid (Fernández de las Heras et al., 2011, 2014).

The breakdown of the steroid ring results from an oxygen-dependent process and subsequent hydrolytic cleavage of rings A and B by a 9,10-secopathway for which the key players are ketosteroid dehydrogenases (KstDs) and a ketosteroid hydroxylase (KSH). KstDs are FAD-dependent enzymes that catalyze the introduction of a double bond between the C1 and C2 atoms in the A ring of 3-ketosteroid substrates (Rohman and Dijkstra, 2019). Different isoforms of KstDs have been found to display different substrate ranges and have different roles that may be strain-dependent (Fernández de las Heras et al., 2012). KSH initiates cleavage of the steroid polycyclic ring by C9a-hydroxylation (Petrusma et al., 2014). KSH is a two-component enzyme consisting of terminal oxygenase (KshA), which performs substrate hydroxylation, and ferredoxin reductase (KshB), which provides electron transfer (van der Geize et al., 2008; Guevara et al., 2017). KSH catalyzes the hydroxylation reaction of AD and ADD to form 9α-hydroxy-AD and 9α-hydroxy-ADD (van der Geize et al., 2002; Petrusma et al., 2009).

There is a significant number of reports on biotransformation of steroids by *Rhodococcus* spp. (Carpova et al., 2011; Nogovitsina et al., 2011; Petrusma et al., 2014; Guevara et al., 2017; Rohman and Dijkstra, 2019; Zappaterra et al., 2021). As model strains for studying the steroid metabolism, *R. erythropolis* SQ1, *R. ruber* Chol-4, *R. jostii* RHA-1, *R. rhodochrous* DSM43269 were described (van der Geize et al., 2002, 2008; Fernández de las Heras et al., 2009, 2012, 2014; Petrusma et al., 2009, 2014; Yam et al., 2010; Mohn et al., 2012; van Oosterwijk et al., 2012; Costa et al., 2013;; Haußmann et al., 2013; Guevara et al., 2017, 2019a; Chen et al., 2020; Baldanta et al., 2021; Zappaterra et al., 2021). In most studies, cholesterol, less commonly β-sitosterol, campesterol, cortisone, and cholate, are used as an initial substrate (Kreit, 2017).

Thus, the identified steroid catabolic capacity of *Rhodococcus* spp. allows using them as model organisms for studying aromatic molecules and steroid biodegradation as well as a biotechnological tool for obtaining steroid pharmaceuticals (Guevara et al., 2019a).

## Production of valuable natural products

**Figure 7:**
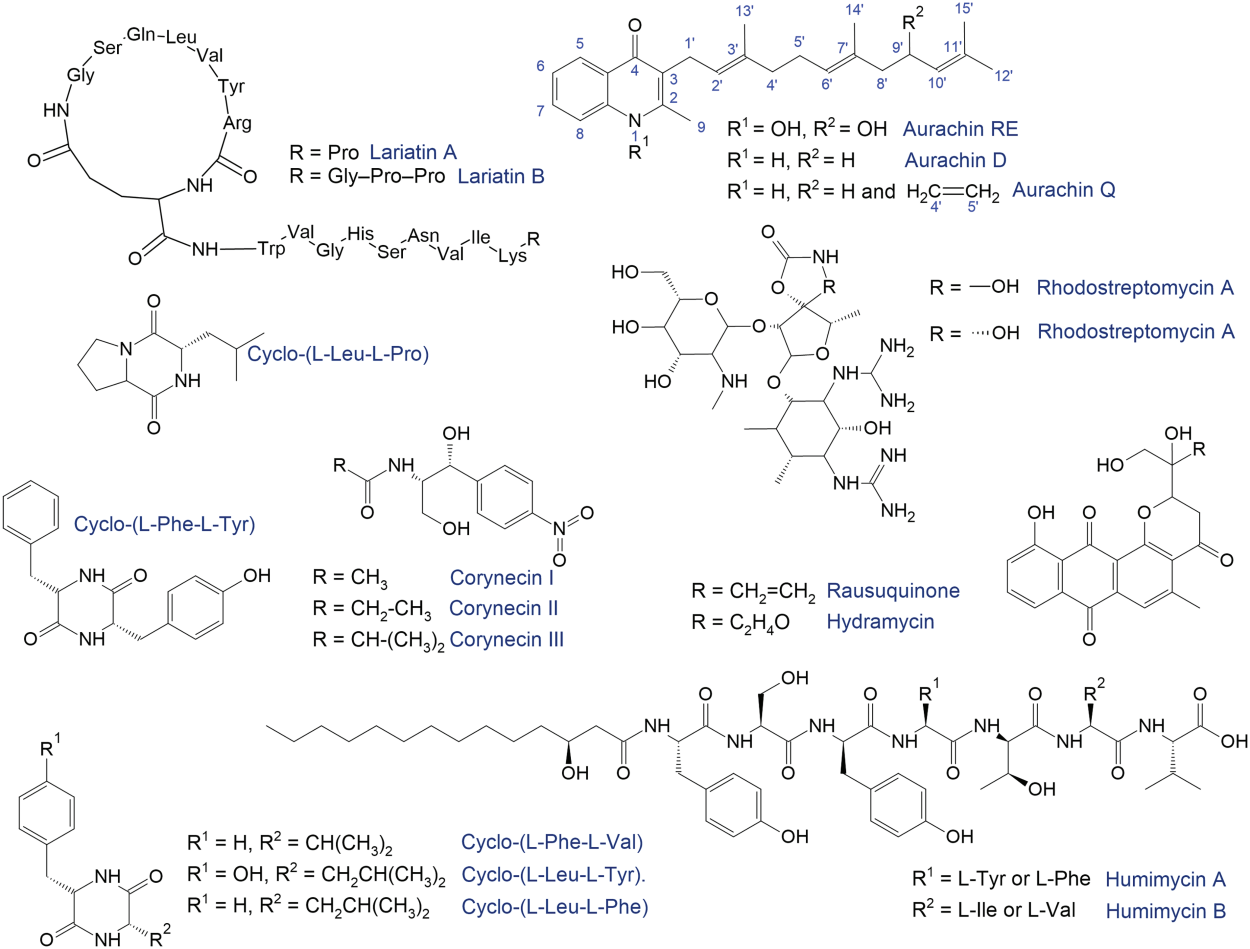
Antimicrobials synthetized by rhodococci (Iwatsuki et al., 2006; Kurosawa et al., 2008; Nachtigall et al., 2010; Chu et al., 2016; Shim, 2016; Undabarrena et al., 2021; Harunari et al., 2022).

In natural habitats, rhodococci produce a huge number of compounds highly valued in the drug discovery and development process (Bosello et al., 2011; Cappelletti et al., 2020; Esposito et al., 2021). In a recent review, Cappelletti et al. (2020) discussed the potential of rhodococci for the production of valuable substances including biosurfactants, bioflocculants, carotenoids, triacylglycerols, polyhydroxyalkanoates, fatty acids, siderophores, antimicrobials, and metal(loid)-based nanostructures. These compounds have significant applications in medicinal and organic chemistry, industry, and pharmaceutical manufacturing. Moreover, synthesis of these compounds ensures the successful competitiveness of rhodococci and comfortable microbial surroundings in their habitats (Ivshina et al., 2021a). Because *Rhodococcus* spp. can produce useful bioactive molecules, they can coexist in mutualistic relationships with other micro- and macroorganisms, providing nutrition to the latter and receiving benefits in return. On the other hand, the synthesis of antimicrobial agents inhibits the growth of certain microorganisms, including pathogenic bacteria. Against the background of antibiotic and multidrug resistance, this property of rhodococci is of great interest to the pharmaceutical industry and is a powerful impetus to the development and discovery of alternative antimicrobial molecules (Sharma et al., 2022; Figure 7).

The search for natural products has traditionally been accompanied by high-throughput screening under laboratory conditions. In particular, the antimicrobial compounds lariatins A and B were found in the culture medium of *Rhodococcus* sp. K01-B0171 using a fermentation-based approach (Iwatsuki et al., 2006). These substances are cyclic peptides with pronounced antimicrobial activity against *Mycobacterium smegmatis*, the causative agent of tuberculosis. *R*. *fascians* 307CO produced aminoglycosides rhodostreptomycin A and B (Kurosawa et al., 2008). The detected antibiotics showed antimicrobial activity against *Streptomyces pandanus*, *Escherichia coli*, *Staphylococcus aureus*, *Bacillus subtilis*, and *Helicobacter pylori*. Interestingly, the ability of strain 307CO to synthesize antibiotics was mediated by horizontal gene transfer from *S*. *padanus*. However, novel rhodostreptomycins A and B differ greatly in structure from other antibiotics produced by *Streptomyces* spp.

Shim (2016) described metabolites produced by the *R*. *rhodochrous* strain belonging to diketopiperazines—the smallest cyclopeptides, which consist of two amino acids. Five diketopiperazines with antimicrobial activities as well as protective effects against post-ischemic myocardial dysfunction have been identified and described. Ward et al. (2018) identified a metabolite produced by *Rhodococcus* sp. MTM3W5.2 inhibiting the growth of *R*. *equi*. The authors proposed a type I polyketide synthase as the main gene involved in the synthesis of this antimicrobial agent.

Rhodococci are capable of synthesizing quinolone antibiotics aurachines. Kitagawa and Tamura (2008) isolated aurachin RE from *R*. *erythropolis* SCM 6824, demonstrating pronounced (0.01–0.1 μg/mL) antimicrobial activity against Gram-positive bacteria *Arthrobacter atrocyaneus*, *Corynebacterium glutamicum*, *Nocardia pseudosporangifera*, *Streptomyces griseus*, and *Rhodococcus erythropolis*. Later, from *Rhodococcus* sp. Acta 2,259, two new aurachins, D and Q, were isolated, showing moderate activity against Gram-positive bacteria, especially *Staphylococcus epidermidis*, *Bacillus subtilis*, and *Propionibacterium acnes* (Nachtigall et al., 2010).

In addition to antibacterial compounds, rhodococci produce antifungal substances. For example, Chiba et al. (1999) identified five tetrapeptides—rhodopeptins Cl, C2, C3, C4 and B5—produced by *Rhodococcus* sp. Mer-N1033 and are active against *Candida albicans* and *Cryptococcus neoformans*.

Culture conditions commonly used in laboratories for microbial growth are typically not optimized for expression of biosynthetic gene clusters which may require induction by unknown signals that originate in the complex natural environments that microbes inhabit (Pan et al., 2019). One of the strategies to overcome this barrier is the one strain many compounds (OSMAC) approach, implying that a single strain can produce different molecules when grown under different environmental conditions (Bode et al., 2002; Pan et al., 2019). Variating the cultivation parameters such as medium composition, pH, temperature, oxygen concentration, metal ions, etc. allows “switching on” the silent or cryptic biosynthetic gene clusters. This approach is especially in demand when exploring natural products from marine actinomycetes. For example, Esposito et al. (2021) identified more than 30 novel glycolipids, particularly succinic saccharide esters with uncommon functional groups (e.g., phenylacetic acid) from *Rhodococcus* sp. I2R. These molecules belong to biosurfactants, which are amphiphilic surface-active compounds containing trehalose (Kuyukina and Ivshina, 2019b; Supplementary Figure 1). *Rhodococcus* biosurfactants may exhibit immune-stimulating, antitumor, and antiviral activities (Kuyukina et al., 2015). So, the identified biosurfactants have antiviral activities against the herpes virus as well as human coronaviruses (HCoV-OC43), and that is undoubtedly of great value in the current situation of the COVID-19 pandemic. In addition, the identified glycolipids have strong antiproliferative activity in the PC3 prostate cancer line (Esposito et al., 2021).

Nowadays, genome sequencing and mining are the latest approaches to search for new bioactive molecules. Computational tools (antiSMASH, PRISM, MIBiG, etc.) have been developed to identify biosynthetic gene clusters and predict the structure of natural products (van Bergeijk et al., 2020). These approaches enabled the identification of a significant number of genes arranged in biosynthetic gene clusters, even in strains that seemed to have been thoroughly studied (van Bergeijk et al., 2020). Analysis of the complete genome of *Rhodococcus* sp. NIOT_ASA1-1 isolated from the marine ascidian *Phallusia nigra* revealed genes encoding the non-ribosomal peptide synthetase and putative polyketide synthase (Meena et al., 2021). High-quality comparative genome mining allowed the identification of a number of biosynthetic gene clusters in *Rhodococcus* sp. H-CA8f involved in producing corynecins (*N*-acyl derivatives of D-threo-*p*-nitrophenylserinol compounds structurally similar to the antibiotic chloramphenicol), which exhibit antimicrobial properties (Undabarrena et al., 2021).

Currently, bacteria from rare or challenging (i.e., nutritional imbalance, waterlogging, hypoxia and anoxia, pollution, salinity, heat, and cold) conditions attract particular interest in searching for biosynthetic genes and novel natural products, which avoids the replication of already known molecules. Stress-tolerant *Rhodococcus* spp. thriving in deserts, permafrost, cave ecosystems, the deep sea, animal and human microbiota are of interest (Ceniceros et al., 2017; Jia et al., 2020a; Meena et al., 2021; Wang et al., 2021; Zamora-Quintero et al., 2022). Thus, polycyclic pluramycin-class polyketides, rausuquinone and hydramycin, were isolated from a deep sea-derived *Rhodococcus* sp. RD015140 (Harunari et al., 2022). Rausuquinone has antimicrobial activity (25–100 μg/mL) against Gram-positive pathogenic bacteria (*Bacillus subtilis*, *Kocuria rhizophila*, and *Staphylococcus aureus*).

The human-associated microbiome represents a source of promising therapeutic molecules (Chu et al., 2016). *Rhodococcus*-produced bacteriocins can be implemented as a potential microbiome-editing tool for precise therapy and prevention of infections (Heilbronner et al., 2021). Bioinformatics prediction from the primary sequence of the gut microbiota followed by chemical synthesis enabled identification of biosynthetic gene clusters found in the genomes of *R*. *equi* and *R*. *erythropolis* responsible for biosynthesis of novel antibiotics named humimycins A and B, which were active against pathogenic *Staphylococcus* and *Streptococcus* species (Chu et al., 2016).

Microbial pigments are also beneficial candidates for medical use, which are less toxic than their synthetic analogues. *Rhodococcus* spp. produce various carotenoid pigments that cause the fawn, yellow-orange, and red color of colonies (Jones and Goodfellow, 2015). The main function of pigments is to protect cells from reactive oxygen species produced by UV radiation and toxic substances (Cappelletti et al., 2020). The pigments extracted from a soil bacterium, *Rhodococcus* sp. SC1, showed antimicrobial activity (MIC 64–256 μg/mL) and antibiofilm-forming (complete inhibition at 32–64 μg/mL) activity against Gram-negative bacteria (*Pseudomonas aeruginosa* and *Escherichia coli*; Çobanoğlu and Yazıcı, 2021).

Another current trend is the use of rhodococci in eco-friendly nano-therapeutics (Alam et al., 2021). *Rhodococcus* spp. are able to bioconvert toxic metal(loid)s generating nanostructures with biomedical, electronic, and environmental applications (Presentato et al., 2018b,c, 2019, 2020). For instance, *R*. *rhodochrous* MOSEL-ME29 produced silver nanoparticles which displayed *in vitro* significant anticancer, antileishmanial, and antiviral activities and were non-toxic to cell cultures. In another study, an *R*. *kroppenstedtii* strain from mangrove soils synthesized gold and silver nanoparticles with antibacterial action against the pathogens—*Vibrio harveyi* and *P*. *aeruginosa* (Kulkarni et al., 2022). In addition, biogenic synthesis of nanoparticles can enhance bioavailability and biodegradation of low concentrations of emerging pollutants (Annamalai and Vasudevan, 2020).

## Quorum-quenching

Quorum quenching (QQ) is an enzyme-dependent alteration or inhibition mechanism of quorum sensing and advantageous antimicrobial treatment (Rémy et al., 2018; Turan and Engin, 2018; Paluch et al., 2020; Zhou et al., 2020). Quorum sensing is cell-to-cell communication through sending and receiving signal molecules called autoinducers and plays a vital role in biofilm formation, pathogenesis and virulence, and antibiotic resistance (Nain et al., 2020). Acetyl-homoserine lactones (AHL) act as auto-inducers in ubiquitous Gram-negative pathogens. In the natural environment, QQ has great evolutionary and ecological significance as a mechanism of interspecific antagonism (Prazdnova et al., 2022). QQ acts by (i) degradation of autoinducers, (ii) blockage of autoinducers’ production, and (iii) interference with autoinducers and their receptors (Grandclément et al., 2016; Turan and Engin, 2018; Paluch et al., 2020). The main AHL-degrading enzymes are lactonases and acylases. Lactonases catalyze the opening of the lactone ring, whereas acylases remove the acyl chain from the homoserine lactone moiety (Billot et al., 2020).

**Figure 8:**
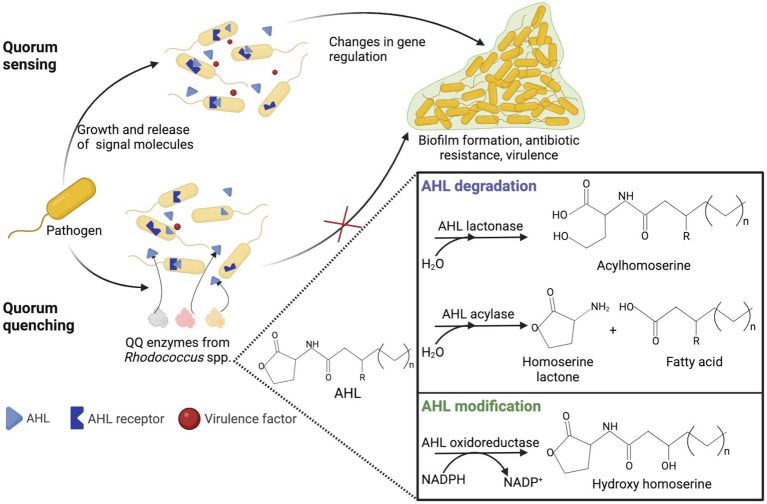
Representation of quorum sensing in Gram-negative bacteria and rhodococcal quorum quenching. AHL, acetyl-homoserine lactone; QQ, quorum quenching.

The use of QQ ensures the microbial balance and prevents the development of bacterial resistance (Barbey et al., 2018). It is known that members of *Rhodococcus* spp. are capable of counteracting biofilm formation through QQ (Uroz et al., 2008; Kwasiborski et al., 2015; Barbey et al., 2018; Kalia et al., 2019; Chane et al., 2020; Bourigault et al., 2021). Rhodococci are of particular interest because they can produce both AHL-degrading enzymes and AHL-modifying enzymes (oxidoreductases), providing an effective QQ against a wide range of AHL (Figure 8). In addition, rhodococci simultaneously exhibit lactonase and acylase activities, a quite rare phenomenon among other bacteria (Prazdnova et al., 2022). The abovementioned indicates the prospects of using *Rhodococcus* QQ in antimicrobial and antivirulence strategies. Additionally, the use of QQ is promising for the control of biofouling of membrane bioreactors as well as the anode in the microbial fuel cell used in wastewater treatment (Oh et al., 2013; Iqbal et al., 2018; Lee et al., 2020; Taşkan and Taşkan, 2021; Güneş and Taşkan, 2022; Shah et al., 2022). The reduced ability to form biofilms and produce extracellular biopolymers enables a significantly enhanced wastewater treatment process, including removal of pharmaceuticals. Nonetheless, a number of researchers highlight the disadvantages of QQ. For example, the suppressive effects of QQ can only be observed with regard to certain bacteria, and such effects are ignored at the level of the bacterial community (Maddela and Meng, 2020).

## Biodegradation of pharmaceutical pollutants

Rhodococci have exceptional xenobiotic-degrading abilities that provide their potential contribution to the bioconversion of pharmaceutical contaminants of various therapeutic classes. Rhodococci represent a pool of microorganisms involved in natural attenuation of pharmaceutical micropollutants. This is evidenced by the data on *Rhodococcus* spp. identification in indigenous pharma-degrading microbiota (Jiang et al., 2017; Ricken et al., 2017; Yang et al., 2020). On the other hand, effective bioremediation techniques for polluted environments should involve microorganisms with enhanced adaptation strategies to environmental stressors (Syed et al., 2021), which raises stress-tolerant *Rhodococcus* spp. to the rank of promising biotechnology tools. For instance, halophilic rhodococci were isolated from the consortium in the air cathode microbial fuel cell for the treatment of pharmaceutical wastewater and generation of bioenergy under high salinity (Pugazhendi et al., 2022). Supplementary Table 1 summarizes information about the ability of rhodococci to degrade drugs from different therapeutic groups.

### Antibiotics

The presence of antibiotics in the environment is recognized as one of the top 10 global public health threats facing humanity (World Health Organization, 2020). Antibiotics were detected in wastewater, soils, sediments, surface water, groundwater, and drinking water in a concentration range of ng/L to hundreds of mg/L (Kovalakova et al., 2020; Yin, 2021; Duan et al., 2022; Junaid et al., 2022). Antibiotic misuse and overuse are the main drivers in the spread of antibiotic-resistant pathogens and genes (Zhang et al., 2021a). The problem has gradually worsened since the beginning of 2020 because of the COVID-19 pandemic and bacterial co-infections and superinfections accelerating the evolution and development of antimicrobial resistance (Afshinnekoo et al., 2021; Lucien et al., 2021; Rodríguez-Baño et al., 2021; Cappelli et al., 2022; Morales-Paredes et al., 2022; Wojcieszyńska et al., 2022). Additionally, discarded face masks pose a serious threat, which, when released into the environment, act as carriers of pollutants, pathogens, antibiotic resistance genes and antibiotics themselves (Lin et al., 2022; Zhou et al., 2022).

Microorganisms are capable of inactivation and mineralization of antibiotics. Nonetheless, knowledge of processes and genes involved in their degradation is sparse (Reis et al., 2020; Sodhi et al., 2021). The study of antibiotic metabolism by rhodococci of ecologically significant species and other antibiotrophs not only provides information about the possible ecological fate of antibiotics and their involvement in biochemical cycles, about the development of bioremediation tools and biological treatment of antibiotic-polluted areas but also expands fundamental knowledge about the protection of susceptible pathogenic microorganisms and the evolution and spread of antimicrobial resistance (Reis et al., 2020).

Rhodococci are known for a set of antibiotic resistance mechanisms, whether efflux pumps, antibiotic-resistance genes encoding drug/metabolite transporters, beta-lactamase related proteins, penicillin-binding proteins, proteins involved in folate biosynthesis, and unique cell wall containing α-alkyl-β-hydroxy fatty acids named mycolic acids (de Carvalho et al., 2014; Orro et al., 2015; Jia et al., 2020b). Moreover, when exposed to antibiotics, *Rhodococcus* spp. may go into a viable but non-culturable state with restricted metabolic activity and function (Yu et al., 2022a).

Depending on the mechanism of action, antibiotics are divided into several categories, specifically aminoglycosides, glycopeptides, ionophores, sulfonamides, beta-lactams, tetracyclines, lincosamides, fluoroquinolones, and diaminopyrimidines (Cycoń et al., 2019).

Sulfonamides inhibit the bacterial enzyme dihydropteroate synthetase, which condenses pteroate and *p*-aminobenzoic acid in the folic acid pathway, thereby blocking bacterial nucleic acid synthesis (Samanta and Bandyopadhyay, 2020). It was rhodococci that were used in the first studies of the bioconversion of sulfonamides by individual bacterial strains. For example, *R*. *rhodochrous* ATCC 13808 cometabolized sulfamethizole (43.4 mg/L) and sulfamethoxazole (31.6 mg/L) by 14 and 20%, respectively, for 12–36 days (Gauthier et al., 2010). The hydroxylated product hydroxy-N-(5-methyl-1,2-oxazol-3-yl) was identified. Larcher and Yargeau (2011) used not only individual strains but also bacterial mixtures. Among the tested rhodococcal strains (*R. equi* ATCC 13557, *R. erythropolis* ATCC 4277, *R. rhodochrous* ATCC 13808, and *R. zopfii* ATCC 51349), significant biodegradation of sulfamethoxazole was noted only in the presence of ATCC 13557. The antibiotic was degraded by 29 and 15% with and without glucose, respectively, for 120 h. Despite a significant increase in biomass and high viability of other rhodococcal strains in the presence of sulfamethoxazole, its depletion was 0–6.6%. This can apparently be explained by the presence of efflux pumps in rhodococci, which provide effective transport of the antibiotic from cells (de Carvalho et al., 2014). Based on the results of inhibition tests, which allow determining the possibility of shared growth of bacterial cultures, the authors then selected two bacterial mixtures. The removal of sulfamethoxazole was, however, about 5% for 300 h. The authors suggested that arylamine N-acetyltransferase, amidase, urethanase, and N-acetyl-phenylethylamine hydrolase were involved in sulfamethoxazole bioconversion.

Since ozonation is a widely used method of wastewater treatment, the authors studied the effect of ozone pre-treatment on the degradation activity of bacteria (Larcher and Yargeau, 2012). It was found that after pre-treatment with O_3_, the microbial consortium consisting of *P. aeruginosa*, *P. putida*, *R. equi*, *R. erythropolis*, and *R. rhodochrous* in the presence of glucose (0.5 g/L) degraded 6 mg/L of sulfamethoxazole by 59% for 300 h. Another selected consortium (*B. subtilis*, *P. putida*, *R. equi*, *R. erythropolis*, *R. rhodochrous*, and *R. zopfii*) converted sulfamethoxazole by 55%. Notably, without O_3_ pre-treatment, the first consortium removed the antibiotic by 40–42%, while the second consortium degraded the antibiotic by 15–31%.

In another research, *Rhodococcus* sp. BR2 isolated from a membrane bioreactor partially transformed 0.5 mM sulfamethoxazole for 400 h. Interestingly, co-cultivation of BR2 with *Microbacterium* sp. BR1 showed both higher conversion rates and shorter lag times than individual cultures, probably caused by syntrophy (Bouju et al., 2012).

In recent years, attention has been paid to emerging bioelectrochemical technologies such as microbial fuel cells (MFCs), microbial electrolysis cells (MECs), and electrochemical membrane biofilm reactors (EMBfRs). Latest studies proved that MFCs can effectively remove antibiotics and reduce the occurrence of ARGs in biofilms (Zhang et al., 2021a). Using acclimatized MFCs, 92.83 ± 1.54% degradation of sulfamonomethoxine (10 mg/L) was achieved. When the antibiotic was introduced into the MFC, this led to a shift from electroactive bacteria to bacteria with dual functions of electricity generation and antibiotic degradation, and members of the genus *Rhodococcus* were found among those bacteria. The metabolites formed during the sulfamonomethoxine degradation did not have pronounced cytotoxicity.

Li et al. (2021) designed an EMBfR to efficiently (> 94.9%) remove sulfadiazine (100 μg/L). The phylum *Actinomycetota* was the most abundant (61%) in the sulfadiazine-degrading microbial community, where *Rhodococcus* spp. accounted for 51%. The removal of sulfadiazine was accompanied by its electrochemical transformation under the influence of the reactive oxygen species formed, the generation of less toxic intermediates and their subsequent bacterial degradation. The above examples of bioelectric transformation of sulfonamides indicated a decrease in the accumulation of ARGs and the consequent dissemination risks.

Fluoroquinolones inhibit DNA gyrase and type IV topoisomerase, resulting in impaired DNA replication and even microbial cell death (Feng et al., 2019). Fluoroquinolones are highly recalcitrant compounds because of the electronegativity and stability of the C-F bond. Only a few cases of degradation of these antibiotics by rhodococci have been documented. Enantioselective biodegradation of fluoroquinolones was investigated by Maia et al. (2018). In the presence of acetate, *Rhodococcus* sp. FP1 cometabolized 70–450 μg/L racemic ofloxacin by 51.9–60.6% for 28 days. At the same time, the (*S*)-enantiomer of ofloxacin (levofloxacin) was transformed more efficiently, which led to the enrichment of the (*R*)-enantiomer in the medium. In relation to another fluoroquinolone, ciprofloxacin, the degradation activity of rhodococci (in particular *Rhodococcus* sp. B30) was not confirmed (Githinji et al., 2011).

Tetracyclines are broad-spectrum antibiotics that inhibit protein synthesis by preventing the attachment of aminoacyl-tRNA to the ribosomal acceptor (A) site (Chopra and Roberts, 2001). *Rhodococcus* spp. strains were detected in agricultural soil supplemented with cow manure (Yeom et al., 2017). Rhodococci showed resistance to oxytetracycline and a mixture of antibiotics (tetracycline + oxytetracycline + sulfathiazole). Under batch cultivation in the nutrient medium, the degradation of sulfathiazole (0.23 mg/kg), tetracycline (17.74 mg/kg), and oxytetracycline (0.87 mg/kg) was 23.53, 35.60, and 66.88%, respectively.

It is known that communication of individual species within microbial consortia is complex and multifaceted. Frequently, monocultures are not capable of metabolizing complex organic pollutants. However, in the community, microorganisms are successful in their synergistic action on the substrate. On the other hand, effective bioconversion of xenobiotics by the microbial community can be explained by the presence of non-degrading bacteria providing fatty acids, ATP, and amino acids to degraders (Liu et al., 2017).

In addition, it is essential to establish the relationship between the antibiotic load and the survival strategy of microorganisms under the combined action of pollutants. Thus, for example, norfloxacin-exposed rhodococci entered the dormant state accompanied by a thickened cell wall and an increased roughness at the morphometric level. While at the metabolic and functional levels, errors during DNA replication, subsequent homologous DNA repair (as evidenced by the increased content of repair proteins RecA, uvrA, UvrB, and UvrC in dormant forms), suppression of transporter proteins, ATP production, and the tricarboxylic acid cycle were observed (Jia et al., 2020b; Yu et al., 2022a). Given the simultaneous presence of many pollutants in the environment, bacterial adaptation to antibiotics reduces the degradation efficiency of other priority pollutants. In this regard, the authors emphasized that the detection of biodegradative genes *in situ* is not sufficient to confirm the ongoing biodegradation processes since the degradation phenotype may not be expressed.

### Hormones

Steroid hormones are biologically active compounds derived from cholesterol that act as chemical messengers in the body. Steroid hormones regulate many physiologic processes, including the development and function of the reproductive system. Steroid hormones are found throughout the environment and pose an increased threat since they act on non-targeted organisms as endocrine disruptors, leading to disturbances in the functioning of the biota at both the organismal and population levels (Abreu et al., 2018; Xia et al., 2019; Myosho et al., 2021; Pironti et al., 2021). Natural androgens (androsterone, epiandrosterone and androsta-1,4-diene-3,17-dione, 4-androstene-3,17-dione, 5α-androstan-17β-ol-3-one), synthetic androgens (17a-boldenone, 17b-boldenone), synthetic progestogens (megestrol acetate, norethindrone and medroxyprogesterone acetate) and natural progestogens (progesterone, 20β-dihydroxy-4-progegnen-3-one, 21α-hydroxyprogesterone, and 17α-hydroxyprogesterone), natural estrogens (estrone (E1), 17β-estradiol (E2) and estriol (E3)), and synthetic estrogens (17α-ethinylestradiol (EE2)) were detected among environmental hormones (Menashe et al., 2020). Complete mineralization of hormones is possible only due to the catalytic activity of microorganisms. Rhodococci demonstrate an outstanding ability to degrade steroid estrogens. It is worth noting that successful survival of rhodococci under steroid contamination is associated with their ability to accumulate steryl esters as spare intracellular components (Holert et al., 2020).

Studies on the biodegradation of hormones by *Rhodococcus* strains were initiated in the early 2000s. Yoshimoto et al. (2004) achieved complete degradation of 100 mg/L of estrogens (E1, E2, E3, and EE2) using strains of *R*. *equi* and *R*. *zopfii*. In the presence of cosubstrate (glucose, adipic acid), members of *R*. *erythropolis* and *R*. *equi* decomposed EE2 (0.5 and 1.4 mg/L) by 39–47% for 12 days (O’Grady et al., 2009). Further, Larcher and Yargeau (2013) reported complete degradation of EE2 (5 mg/L) by *R*. *rhodochrous* ATCC 13808 cells in 48 h. Complete decomposition of androgen testosterone (1 mg/mL) was achieved within 2 days using non-growing cells of *R*. *equi* ATCC 14887. Among the end products of biodegradation, 3-hydroxy-9,10-secoandrosta-1,3,5(10)-triene-9,17-dione was identified and apparently further decomposed into carbon dioxide and water (Kim et al., 2007).

To enhance the biodegradation of hormones, immobilization and encapsulation of rhodococci were employed. For instance, alginate-immobilized *Rhodococcus* sp. strain JX-2 cells completely decomposed E2 (30 mg/L) in a mineral medium under suboptimal conditions (pH below 6 or above 9, temperature below 20 or above 35°C) as well as in natural wastewater and cow manure samples (Liu et al., 2016). Menashe et al. (2020) studied the degradation of EE2 (0.5 mg/L) using the Small Bioreactor Platform macro-encapsulation method (entrapment of bacteria in cellulose acetate microfiltration membrane capsules). The authors encapsulated two strains, *R. zopfii* ATCC 51349 and *P. putida* ATCC 700007, capable of degrading EE2 as the sole carbon and energy source by 90.4 and 72.7%, respectively. In secondary wastewater effluent, ATCC 51349 degraded this estrogen by 84.2% over the same period. The encapsulation method used in the work can be applied at wastewater treatment plants as an effective biological bioaugmentation approach, which allows controlling and maintaining the degrading biomass at an efficient level.

Currently, studies on the genetic mechanisms of estrogen bioconversion are ongoing. In this context, the whole-genome sequencing of *R*. *equi* strain DSSKP-R-001, which is an active degrader of E1, E2, and EE2, enabled to detect genes that presumably encode KstDs, KSHs, ChOx, and other enzymes of steroid degradation (Zhao et al., 2018; Wang et al., 2019; Tian et al., 2020). Bioinformatics analysis of the whole-genome sequence of *Rhodococcus* sp. P14 (E2, E3 and testosterone biooxidizer) revealed the presence of a key degradation enzyme—a short-chain dehydrogenase 17ß-HSDx (Ye et al., 2019).

A recent study by Hsiao et al. (2021) showed the ability of *Rhodococcus* sp. B50 to degrade E1, E2, E3, testosterone or cholesterol as the only carbon and energy source. Modification of the E1 molecule followed the 4,5-seco pathway and was accompanied by the formation of at least 4 products, including pyridinestronic acid and 3aa-H-4a(3’-propanoate)-7ab-methylhexahydro-1,5-in-danedione. Whole-genome sequencing and bioinformatics analysis of *Rhodococcus* sp. B50 allowed studying and identifying a gene cluster located on the circular megaplasmid and responsible for estrogen degradation. This cluster harbors two genes, *aedA* (cytochrome P450 monooxygenase) and *aedB* (4-hydroxyestrone 4,5-dioxygenase), with a key role in activating and cleaving A-ring, as confirmed by side-directed mutagenesis. The authors consider that the detected metabolites and genes can be used as biomarkers of ecosystem pollution by estrogens. Another set of degrading enzymes was identified in *R*. *equi* ATCC13557 (Harthern-Flint et al., 2021). These are cytochrome P450 monooxygenase (*oecB*), extradiol dioxygenase (*oecC*), and 17ß-hydroxysteroid dehydrogenase (*oecA*). In addition, gene clusters encoding the metabolism of other steroids (e.g., cholesterol) were found.

Despite numerous reports on the degradation of estrogens and androgens by rhodococci, there is only one report on the bioconversion of progestogens. Yu et al. (2018) isolated *Rhodococcus* sp. strain HYW from the activated sludge of the treatment plant capable of progesterone degradation as the only source of carbon and energy. *Rhodococcus* sp. HYW degraded progesterone (500 μg/L) by 99% within 1 h. Importantly, HYW-bioaugmentation of the activated sludge enabled the enhancement of the progesterone bioconversion process. The authors described two metabolic pathways in non-augmented and augmented activated sludge. Whole-genome sequencing of *Rhodococcus* sp. HYW revealed five steroid-degrading genes encoding 3-ketosteroid-9-alpha-hydroxylase (KSH), 3-ketosteroid delta (1)-dehydrogenase (KstD), 3α-hydroxysteroid dehydrogenase (3α-HSD), cholesterol oxidase (ChOx), and monooxygenase (MO).

### Antispasmodic agents

Antispasmodic agents such as belladonna, mebeverine, pinaverine, dicyclomine, hyoscyamine, papaverine, drotaverine, trimebutine, etc. are drugs designed to relieve spasm of the smooth muscles of bronchi, blood vessels, digestive tract, biliary, and urinary tracts. Information on environmental detection and ecotoxicity of these pharmaceutical substances is extremely insufficient. Drotaverine (*syn*. No-Spa®), an antispasmodic, vasodilator, and myotropic agent highly demanded in Asia, Russia, and Eastern Europe, was found in Saint Petersburg’s wastewater in concentrations up to 452 ng/L (HELCOM, 2014). Ivshina et al. (2012) conducted a series of experiments to study the ability of rhodococci to biodegrade drotaverine employing bioresources of the Regional Specialised Collection of Alkanotrophic Microorganisms (acronym IEGM, WDCM 768, http://www.iegmcol.ru). Based on the research results, *R*. *rhodochrous* IEGM 608 was capable of biodegradation of this pharmaceutical (200 mg/L) as the only carbon and energy source. The authors used various methods to enhance biodegradation: preincubation of bacteria, additional energy substrates, and immobilization on solid carriers (pine sawdust). It enabled them to achieve complete degradation of drotaverine within 30 days. The pathways of bacterial drotaverine decomposition accompanied by the formation of 3,4-diethoxybenzoic acid (protocatechuic acid) derivatives were proposed. Further, the authors studied drotaverine degradation by rhodococci at different physiological states. It is well known that many bacteria can withstand stressors such as cold, desiccation, and xenobiotics by going into dormant (metabolically inactive) states. Resuscitation of degraders in the dormant state may accelerate the rate of xenobiotic degradation in the environment (Yu et al., 2022b). Compared with vegetative cells, reactivated cyst-like dormant cells of *R*. *ruber* IEGM 346 demonstrated increased degrading activity towards drotaverine as the only carbon and energy source and under co-oxidation (glucose), including suboptimal (35 ± 2°C) growth conditions (Ivshina et al., 2015).

### Analgesics

Paracetamol, a *p*-aminophenol derivative, is a commonly used and most frequently detected analgesic compound in surface water (up to 227 μg/L; Wilkinson et al., 2022). According to Ivshina et al. (2006), *R*. *erythropolis* IEGM 77 and *R*. *erythropolis* IEGM 767 transformed paracetamol to form pyrocatechol, hydroquinone, and benzoquinone. The maximum loss of paracetamol as a substance was 86% for 20 days, and complete biodegradation of paracetamol as tablets was observed after five days. The use of rhodococci immobilized in poly(vinyl alcohol) cryogel reduced the duration of the process to three days.

In addition, Akay and Tezel (2020) isolated 28 paracetamol-degrading strains from soil on a selective medium; of them, 22 strains belonged to *R*. *erythropolis*. The authors studied the acetaminophen degradation kinetics and found that *R*. *erythropolis* BIOMIG-P19 degraded 1, 10, 100, and 500 mg/L for 6 to 24 h. The biodegradation of paracetamol begins with the hydrolysis of the amide bond by aryl acylamidase, leading to acetic acid and 4-aminophenol being formed.

### Nonsteroidal anti-inflammatory drugs

Nonsteroidal anti-inflammatory drugs (NSAIDs) are a group of medicines widely used in medical and veterinary practice that have analgesic, antipyretic, and anti-inflammatory effects and reduce pain, fever, and inflammation. These drugs are among the most frequently and widely detected in the environment (Koumaki et al., 2017; Izadi et al., 2020; Mejía-García et al., 2020; Praveenkumarreddy et al., 2021; Rastogi et al., 2021; Mohd Hanafiah et al., 2022). According to aus der Beek et al. (2016), diclofenac, ibuprofen, naproxen, and acetylsalicylic acid are among the top 10 most common pharmaceutical pollutants in aquatic ecosystems.

Accumulation of NSAIDs in the environment endangers non-target organisms and human health (Oaks et al., 2004; Gamarra et al., 2015; Schmidt and Redshaw, 2015; Mezzelani et al., 2016; Horie et al., 2019; Izadi et al., 2020; Parolini, 2020; Madikizela and Ncube, 2021; Moreno-Opo et al., 2021).

Employing the bioresources of the IEGM collection, we have launched a project to search for effective biocatalysts for the degradation of pharmaceutical substances from the NSAID group. In the course of our own research, using *R*. *ruber* IEGM 346 isolated from wastewater, we showed for the first time the ability of rhodococci to effectively degrade a polycyclic NSAID diclofenac via breaking the C-N bond and opening the aromatic ring in the diclofenac molecule with the formation of fumaryl acetoacetic acid and its hydrolysis products—non-toxic and non-hazardous to the environment (Ivshina et al., 2019). In aquatic ecosystems, a pharmaceutical cocktail of different therapeutic groups, including NSAIDs, is commonly found (Dasenaki and Thomaidis, 2015; Nebot et al., 2015). In the study of diclofenac in a mixture with ibuprofen and meloxicam, it was found that a cocktail of these substances, even at lower concentrations, causes a greater inhibitory effect on *R*. *ruber* IEGM 346 cells than these compounds separately. The obtained data are consistent with other studies demonstrating that a pharmaceutical cocktail in lower concentrations causes greater ecotoxicological damage than individual compounds (Barra Caracciolo et al., 2015).

*R*. *cerastii* IEGM 1278, a plant-associated strain, carried out complete biodegradation of 100 μg/L and 100 mg/L of ibuprofen for 30 and 144 h in the presence of 0.1% *n*-hexadecane, respectively (Ivshina et al., 2021b). It is worth noting, there is ever mounting number of the basic studies of stress-tolerant plant-associated microorganisms and plant-microbe interaction as well as applied aspects, particularly rhizoremediation, as an effective strategy for xenobiotic biodegradation (Vergani et al., 2019; González-Benítez et al., 2021; Kuhl et al., 2021; Singha and Pandey, 2021; Zeng et al., 2022). *R*. *josii* IEGM 60 was isolated from oil-polluted soil and degraded acetylsalicylic acid (250 mg/L) in the form of a pharmaceutical substance and tablets within 11 and 6 days, respectively (Khrenkov et al., 2020). Recently, *R*. *erythropolis* IEGM 746 and *R*. *rhodochrous* IEGM 63 were selected as being capable of partial cometabolization of 100 mg/L ketoprofen and naproxen, respectively (Bazhutin et al., 2022; Ivshina et al., 2022).

**Figure 9:**
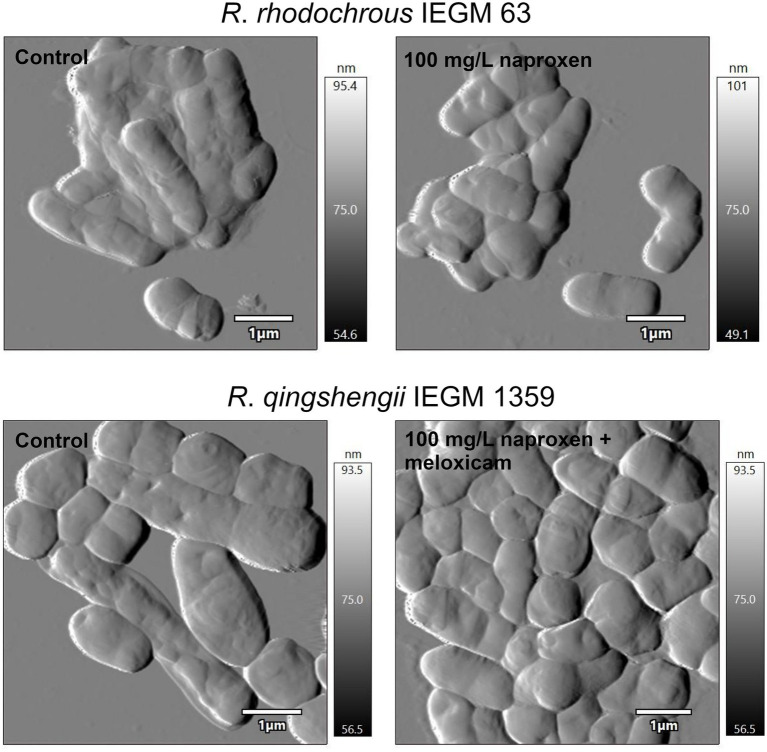
Atomic force microscopy images of rhodococci exposed to nonsteroidal anti-inflammatory drugs (NSAIDs). Cells were cultivated in a mineral salt medium supplemented with 10 mg/L naproxen or 10 mg/L naproxen +10 mg/L meloxicam for 7 days. When exposed to NSAIDs, rhodococci form multicellular aggregates and change their shape and size. This contributes to a modification in the surface area-to-volume ratio, which is of great importance in the adaptation of bacteria to xenobiotics (Ivshina et al., 2022).

The universal adaptation mechanisms of *Rhodococcus* in response to the above-mentioned NSAIDs present were described: *Rhodococcus* shift from single- to multicellular lifeforms (i.e., the formation of bacterial aggregates) accompanied by a marked morphological anomaly of cells (e.g., changed shape and size, reduced surface-to-volume ratio as well as changed parameters of cell surface roughness; Figure 9), a zeta-potential (i.e., bacterial surface charge) shift to more negative values and decreased permeability of cell membranes. We consider the obtained data as mechanisms of *Rhodococcus* adaptation and, consequently, of their increased resistance to the effects of pharmaceutical pollutants and their metabolites (Ivshina et al., 2019, 2021b, 2022).

### Other pharmaceuticals

*R*. *rhodochrous* ATCC 13808 degraded an antiepileptic agent carbamazepine (9.5 mg/L) by 20% for 28 days (Gauthier et al., 2010). Thelusmond et al. (2019) established a possible contribution of rhodococci to bioconversion of resistant pharmaceutical pollutants, i.e., carbamazepine, triclocarban (antibacterial) and triclosan (antiseptic) in the soil microbiota. *R*. *rhodochrous* BX2 isolated from activated sludge could degrade up to 76.8% of 10 mg/l triclocarban as the only carbon source after 5 days (Li et al., 2022). The identified key enzymes included amidase, able to catalyze the conversion of triclocarban to 3,4-dichloroaniline and 4-chloroaniline, and a novel phenol hydroxylase responsible for 4-chloroaniline conversion to 4-chlorocatechol.

Induced oxygenase enzyme systems allow the attainment of high rates of degradation and transformation of organic substrates. Cells of *R*. *jostii* RHA1 pre-grown in mineral salt medium with propane or biphenyl and in LB medium with dicyclopropylketone degraded 5 mg/L of triclosan by 35, 64, and 63%, respectively, for 3 days (Lee and Chu, 2013). The hydrocarbons induced monooxygenases (propane monooxygenases and alkane-monooxygenase) or dioxygenases (e.g., biphenyl dioxygenase). These enzymes have broad substrate specificities and can oxidize a huge variety of complex organic compounds (Gravouil et al., 2017). Biphenyl-grown rhodococci degraded triclosan through meta-cleavage to form four products (2,4-dichlorophenol, 2-chlorohydroquinone, monohydroxy-triclosan, and dihydroxy-triclosan).

## Conclusion and prospects

Overall, *Rhodococcus* spp. can be considered as active natural agents with high transformation and degradation activity towards pharmaceutical compounds (Supplementary Figure 2). Rhodococci are frequently utilized in the biocatalysis of pharmaceutical precursors and novel drug development since they have a powerful set of enzymes (oxidoreductases, lyases, and hydrolases). Pharmaceutical precursor biosynthesis began by implementation of whole cells, then the focus shifted to isolated enzymes and heterologous expression. The use of such *Rhodococcus* biocatalysts made it possible to obtain important building blocks for the synthesis of anticancer, antiviral, anti-inflammatory, lipid-lowering drugs, hormones, and antidepressants in high yields. However, the development of commercially viable biocatalysts requires a lot of time, which is incompatible with the increasing dynamic growth of the pharmaceutical industry. For this purpose, studies are carried out on effective genetic toolkits for creating super-productive (i.e., genetically modified bacterial cells that produce valuable chemical products with a multiple increase in production output compared to wild strains and in an environmentally friendly way) microbial cell factories. The development of precise genome editing techniques such as CRISPR/Cas9 and CRISPRi will help *Rhodococcus* spp. become an efficient platform for the production of valuable compounds (DeLorenzo et al., 2021; Liang and Yu, 2021).

Basic research involving individual *Rhodococcus* strains in pharmaceutical biodegradation processes is currently being conducted. The efficient degraders of analgesics, antibiotics, hormones, and nonsteroidal anti-inflammatory drugs were reported, and rhodococcal metabolizations of these pharmaceuticals were studied. However, *in situ* decomposition of pharmaceutical substances and the metabolic behavior of *Rhodococcus* spp. within communities of microbes that are active in biodegradation are still unclear. The relationships of microorganisms in the natural environment are complex and multifaceted; they are not reproduced in laboratory conditions. It is difficult to take into account interspecific metabolic interactions of rhodococci, extracellular metabolite exchanges, and cross-feeding interactions with non-degrading bacteria (Liu et al., 2019; Huang et al., 2020). In addition, the presence of other pollutants in the environment and their impacts on the process of pharmaceutical degradation have not been fully studied (Xu et al., 2021). Also, it is crucial to understand how pharmaceutical pollutants affect rhodococci and other microbes, which are a natural system of “primary response” to xenobiotic load in open ecosystems. For instance, the occurrence of pharmaceuticals in aquatic or soil ecosystems may lead to reduced biodiversity, changes in biomass composition, biochemical reactions, metabolic and enzymatic activity of a microbiological community, to the suppression of local (native) species and, finally, to the transformation of key ecological processes by synergistic and additive effects of other ecotoxicants and their metabolites (Rosi-Marshall et al., 2013; Aguilar-Romero et al., 2020; Gomes et al., 2020; Crampon et al., 2021; McLain et al., 2022; Pashaei et al., 2022).

Under challenging conditions (i.e., nutritional imbalance, waterlogging, hypoxia and anoxia, pollution, salinity, heat, and cold), naturally occurring rhodococci produce numerous valuable compounds that have medical benefits. These are antimicrobials, biosurfactants, pigments, nanoparticles, and quorum-quenching enzymes. Nevertheless, it is not always possible to identify such chemicals and obtain them using fermentation-based techniques. The selective pressures of various suboptimal environmental conditions on microorganisms and competitive relationships switch on biosynthetic gene clusters that are “silent” in standard laboratory conditions. To activate biosynthetic gene clusters, it is promising to use genetic engineering tools, such as development of heterologous expression hosts, co-expression of regulatory genes, use of transcription factor decoys, refactoring of biosynthetic gene clusters, and knockouts of the core genes of “constitutively” produced compounds (Chu et al., 2016; Gren et al., 2021).

The UN General Assembly proclaimed 2022 the International Year of Basic Sciences for Sustainable Development (IYBSSD 2022). The fundamental tasks touched upon in this review will be solved through active implementation of advanced approaches, such as genomics, metagenomics, proteomics, transcriptomics, and metabolomics (Chen et al., 2014; Henson et al., 2018; Sengupta et al., 2019; Pacwa-Płociniczak et al., 2020; Donini et al., 2021; Mishra et al., 2021; Táncsics et al., 2021; Firrincieli et al., 2022). The use of such “omics technologies” allows us to obtain a comprehensive view of rhodococcal populations, their mechanisms of interaction with pharmaceuticals, their metabolic machinery, evolution and adaptation, genetic regulation and molecular biological aspects (Chevrette et al., 2020; González-Benítez et al., 2021; Mishra et al., 2021; Undabarrena et al., 2021).

It is necessary to create and maintain collections of rhodococcal strains, pan-genome, and metagenome databases, which will entail the acquisition of a huge array of biological data—their analysis and interpretation will assist in obtaining completely new systemic knowledge about bacterial phenomena and processes. As for biological big data processing, difficult to process and interpret, metabolic engineering, metabolic flux determination, enzyme design, etc., it seems reasonable to involve artificial intelligence (machine learning, neural networks, and deep learning; Nagaraja et al., 2020; Kim et al., 2021; Peng et al., 2021; Jang et al., 2022). This will allow us, firstly, to identify previously unexplored therapeutically valuable substances; secondly, to establish detailed pathways of pharmaceutical biotransformation and biodegradation; thirdly, to optimize the conditions (media composition, additional growth substrates, etc.) of biodegradation and biocatalysis. Fourthly, to design a metabolic pathway of new pharmaceuticals, i.e., non-natural compounds, which will enable evaluating the environmental risks of novel molecules in development and minimize unintended and undesirable implications for the natural environment (design by benign concept).

## Author contributions

II conceived and designed the review, coordinated the work, reviewed and edited the manuscript. ET prepared the original project. GB raised issues and revised the manuscript. All authors contributed to the article and approved the submitted version.

## Funding

The study was fulfilled under the State Assignment (АААА-А-19-119112290008-4), the Russian Science Foundation grant (no. 21-14-00132).

## Conflict of interest

The authors declare that the research was conducted in the absence of any commercial or financial relationships that could be construed as a potential conflict of interest.

## Publisher’s note

All claims expressed in this article are solely those of the authors and do not necessarily represent those of their affiliated organizations, or those of the publisher, the editors and the reviewers. Any product that may be evaluated in this article, or claim that may be made by its manufacturer, is not guaranteed or endorsed by the publisher.
