# Supplementary material for: Rhodococcus strains as a good biotool for neutralizing pharmaceutical pollutants and obtaining therapeutically valuable products: Through the past into the future
Source: Front Microbiol. 2022 Sep 29;13:967127. doi: 10.3389/fmicb.2022.967127 (PMC9557007; doi:10.3389/fmicb.2022.967127)
Supplement: Supplementary file 1 [file Table_1.PDF]

Supplementary Table 1. Biodegradation of pharmaceuticals by rhodococci.

Therapeutic class	Compound	Concentration	Biodegradation	Biodegrader	Biodegradation conditions	Metabolites	Reference
Antibiotics	Sulfamethoxazole	31.6 ppm	20%, 36 d	*R. rhodochrous* ATCC 13808	Minimum mineral salt media (MMSM) + 3 g/L glucose	hydroxy-N-(5-methyl-1,2-oxazol-3-yl)ben-zene-1-sulfonamide	(Gauthier et al., 2010)
		6 mg/L	15%, 120 h	*R. equi* ATCC 13557	MMSM	-	(Larcher and Yargeau, 2011)
			29%, 120 h		MMSM + 0.5 g/L glucose	Unknown metabolite	
			3.0–4.6% 300 h	*P. aeruginosa* PA01, *P. putida* ATCC 12633, *R. equi* ATCC 13557, *R. erythropolis* ATCC 4277, *R. rhodocrous* ATCC 13808	MMSM	-	
			4.8–5.1% 300 h		MMSM + 0.5 g/L glucose	Unknown metabolite	
			1.5–2.6% 300 h	*B. subtilis* ATCC 6051, *P. putida* ATCC 12633, *R. equi* ATCC 13557, *R. erythropolis* ATCC 4277, *R. rhodocrous* ATCC 13808, *R. zopfii* ATCC 51349	MMSM	-	
			4.7–5.3% 300 h		MMSM + 0.5 g/L glucose		
			42% 300 h	*P. aeruginosa* PA01, *P. putida* ATCC 12633, *R. equi*	MMSM	-	

Therapeutic class	Compound	Concentration	Biodegradation	Biodegrader	Biodegradation conditions	Metabolites	Reference
			40% 300 h	ATCC 13557, *R. erythropolis* ATCC 4277, *R. rhodocrous* ATCC 13808	MMSM + 0.5 g/L glucose		(Larcher and Yargeau, 2012)
			47% 300 h		Ozone-pretreatment, MMSM		
			59% 300 h		Ozone-pretreatment + 0.5 g/L glucose		
			15% 300 h	*B. subtilis* ATCC 6051, *P. putida* ATCC 12633, *R. equi* ATCC 13557, *R. erythropolis* ATCC 4277, *R. rhodocrous* ATCC 13808, *R. zopfii* ATCC 51349	MMSM		
			31% 300h		MMSM + 0.5 g/L glucose		
			55% 300 h		Ozone-pretreatment, MMSM		
			20% 300h		Ozone-pretreatment + 0.5 g/L glucose		
		0.5 mM	≈40% 300 h	*Rhodococcus* sp. BR2	Mineral salts medium	-	(Bouju et al., 2012)
			≈50% 300 h	*Microbacterium* sp. BR1, *Rhodococcus* sp. BR2			
	Sulfamethizole	43.4 ppm	14%, 12 d	*R. rhodochrous* ATCC 13808	Minimum mineral salt media + 0.5 g/L of yeast extract + 3 g/L glucose	-	(Gauthier et al., 2010)

Therapeutic class	Compound	Concentration	Biodegradation	Biodegrader	Biodegradation conditions	Metabolites	Reference
	Sulfamonomethoxine	10 mg/L	92.83%	Mixed bacterial culture collected from the anode effluent of microbial fuel cells (MFCs). Sulfamonomethoxine-degrading genera: *Cupriavidus*, *Rhodococcus*, *Sphaerochaeta*, and *Cloacibacillus*	Two-chamber MFC reactor	4 metabolites: P127 (m/z 217.1070), P126 (m/z 126.0663), P173 (m/z 173.9852), and P159 (m/z 159.0127)	(Zhang et al., 2021)
		9 mg/L	75.88%				
		8 mg/L	61.42%,				
		7 mg/L	55.37%,				
		6 mg/L	40.25%,				
		5 mg/L	35.41%,				
		4 mg/L	28.82%,				
	Sulfadiazine	100 μg/L	>94.9% 80 d	Activated sludge from a membrane bioreactor located. *Rhodococcus* (51%) was the most dominant genus.	Electrochemical membrane biofilm reactor	Electrochemical oxidation products: Pyrimidin-2-yl)sulfonic acid (EP1) and aniline (EP2), 4-nitro sulfadiazine (EP3) and EP4 (m/z 318).<br><br>Biodegradation products: BP1 (m/z 202), its hydroxylated product (BP2, m/z 218), BP3 (m/z 202), its	(Li et al., 2021)

Therapeutic class	Compound	Concentration	Biodegradation	Biodegrader	Biodegradation conditions	Metabolites	Reference
						acetylated product (BP4, m/z 243)	
	Sulfathiazole	0.23 mg/kg	23.53% 13 d	Antibiotic resistant bacteria from farmland soil	Nutrient broth medium	-	(Yeom et al., 2017)
	Racemic ofloxacin	450 µg/L	39.3% of (*S*)-ofloxacin, 28 d	*Rhodococcus* sp. FP1	Batch mode: mineral salts medium	-	(Maia et al., 2018)
			60.6% of (*S*)-ofloxacin, 28 d		Batch mode: mineral salts medium + 5.9 mM acetate		
		150 µg/L	1.2% of (R)-ofloxacin<br><br>4.1% of (S)-ofloxacin 28 d		Batch mode: mineral salts medium		
			≈50% of (S)-ofloxacin 28 d		Batch mode: mineral salts medium + 5.9 mM acetate		
		70 µg/L	7.4% of (R)-ofloxacin<br><br>4.8% of (S)-ofloxacin, 28 d		Batch mode: mineral salts medium		

Therapeutic class	Compound	Concentration	Biodegradation	Biodegrader	Biodegradation conditions	Metabolites	Reference
			51.9% of (S)-ofloxacin		Batch mode: mineral salts medium + 5.9 mM acetate		
	Tetracycline	17.74 mg/kg	35.6% 13 d	Antibiotic resistant bacteria from farmland soil	Nutrient broth medium	-	(Yeom et al., 2017)
	Oxytetracycline	0.78 mg/kg	66.8% 13 d				
Antimicrobial additives	Triclocarban	10 mg/L	100% after 5 d	*R. rhodochrous* BX2	Modified mineral salt medium (MMSM) used	3,4-dichloroaniline, 4-chloroaniline, 4-chlorocatechol, 3-chloro-cis-cis-muconic acid, muconolactone, β-ketoadipic acid	(Li et al., 2022)
	Triclosan	5 mg/L	35% after 3 d	*R. jostii* RHA1	NMS medium + resting cells, cells were pre-grown in mineral salt medium with propane	-	(Lee and Chu, 2013)
			64% after 3 d		NMS medium + resting cells, cells were pre-grown in mineral salt medium with biphenyl	2,4-dichlorophenol, 2-chlorohydroquinone, monohydroxy-triclosan, and	

Therapeutic class	Compound	Concentration	Biodegradation	Biodegrader	Biodegradation conditions	Metabolites	Reference
						dihydroxy-triclosan	
			63% after 3 d		NMS medium + resting cells, cells were pre-grown in LB medium with dicyclopropylketone	-	
Hormones	Estrone (E1)	30 mg/L	98.6% at 48 h	*R. equi* DSSKP-R-001	Mineral basal medium	-	(Tian et al., 2020)
		100 mg/L	99% 24 h	Individual strains *R. equi* Y 50155, Y 50156, Y 50157 isolated from activated sludge in a wastewater treatment plant	Modified mineral Dominic and Graham's (MDG) culture medium	-	(Yoshimoto et al., 2004)
			100% 24h	*R. zopfii* Y 50158 isolated from activated sludge in a wastewater treatment plant			
			100% 30 h	*Rhodococcus* sp. B50 isolated from garden soil	Resting cell biotransformation in a mineral medium	4-hydroxyestrone, meta-cleavage product, pyridinestrone acid, 3aa-H-4a(3'-propanoate)-7ab-methylhexahydr	(Hsiao et al., 2021)

Therapeutic class	Compound	Concentration	Biodegradation	Biodegrader	Biodegradation conditions	Metabolites	Reference
						o-1,5-in-danedione	
		200 mg/L	90% 120 h	*Rhodococcus* sp. ED7 isolated from agricultural soil	Sterilized inorganic salt medium	-	(Kurisu et al., 2010)
	17β-estradiol (E2)	5 mg/L	80% 18 h	*R. equi* ATCC13557	Mineral salts medium	-	(Harthern-Flint et al., 2021)
		5, 10, 20, 30, 40, 50 mg/L	>80% 7 d	*Rhodococcus* sp. JX-2 isolated from activated sludge	Free cell biotransformation in a mineral salt medium	-	(Liu et al., 2016)
		10, 20, 30, 40, 50, 60, 70 mg/L	>80% 7 d		Alginate-immobilized cell biotransformation in a mineral salt medium under pH 6.0–8.0 and 20–35 °C temperatures		(Liu et al., 2016)
		30 mg/L	94% 7 d		Free cell biotransformation in a mineral salt medium		(Liu et al., 2016)
			≈95% 7 d		Alginate-immobilized cell biotransformation in a mineral salt medium under pH <6.0 or >8.0 either		(Liu et al., 2016)

Therapeutic class	Compound	Concentration	Biodegradation	Biodegrader	Biodegradation conditions	Metabolites	Reference
					below 20 or above 35 °C		
			100% 96 h	*R. equi* DSSKP-R-001	Mineral basal medium	Proposed pathway from transcriptome analysis. E1; 3-Hydroxy-4,5-9,10-disecoestrane-1; 2-Diene-5,9,17-trione-4-oic; (Z)-7a-methyl-4-(prop-1-en-1-yl)hexahydro-1H-indole-1,5-(4H) - Diketone	(Tian et al., 2020)
		39.78 ng/L	64.4% 7 d	*Rhodococcus* sp. JX-2 isolated from activated sludge	Alginate-immobilized cell biotransformation in natural sewage	-	(Liu et al., 2016)
		43.35 ng/L	100% 7 d				
		55.59 ng/L	73.5% 7 d				
		50 mg/L	86% 96 h	*R. equi* DSSKP-R-001	Inorganic minimal salt medium (MSM)	-	(Wang et al., 2019)
			94% 96 h	Mixed culture of *R. equi* DSSKP-R-001 and *Comamonas testosteroni* QYY20150409		Two pathways of E2 metabolisation through hydroxylation of E1 (4-OH-E1 and 16-OH-product),	

Therapeutic class	Compound	Concentration	Biodegradation	Biodegrader	Biodegradation conditions	Metabolites	Reference
						cleavage of 4-OH-E1 between C-4 and C-5 and hydroxylation of E1 at C-9α	
		100 mg/L	100% 24h	*R. zopfii* Y 50158 isolated from activated sludge in a wastewater treatment plant	Modified mineral Dominic and Graham's (MDG) culture medium	-	(Yoshimoto et al., 2004)
			99% 24 h	Individual strains *R. equi* Y 50155, Y 50156, Y 50157 isolated from activated sludge in a wastewater treatment plant			
		100 mg/L	100% 40 h	*Rhodococcus* sp. B50 isolated from garden soil	Resting cell biotransformation in a mineral medium	-	(Hsiao et al., 2021)
		146.31 µg/kg	87.1% 7 d	*Rhodococcus* sp. JX-2 isolated from activated sludge	Alginate-immobilized cell biotransformation in cow dung	-	(Liu et al., 2016)
		174.01 µg/kg	81.3% 7 d				
		200 mg/L	>90% 120 h	*Rhodococcus* sp. ED7 isolated from agricultural soil	Sterilized inorganic salt medium	E1	(Kurisu et al., 2010)
		500 mg/L	100% 100h	*R. zopfii* Y 50158 isolated from activated sludge in a wastewater treatment plant	10-liter jar fermentor, MDG medium + 10 g/L glucose	-	(Yoshimoto et al., 2004)

Therapeutic class	Compound	Concentration	Biodegradation	Biodegrader	Biodegradation conditions	Metabolites	Reference
	Estriol (E3)	100 mg/L	95% 24 h	Individual strains *R. equi* Y 50156, Y 50157 isolated from activated sludge in a wastewater treatment plant	Modified mineral Dominic and Graham's (MDG) culture medium	-	(Yoshimoto et al., 2004)
		100 mg/L	72% 24 h	Individual strain *R. equi* Y 50155 isolated from activated sludge in a wastewater treatment plant	Modified mineral Dominic and Graham's (MDG) culture medium	-	(Yoshimoto et al., 2004)
			100% 24h	*R. zopfii* Y 50158 isolated from activated sludge in a wastewater treatment plant			
		100 mg/L	≈90% 50 h	*Rhodococcus* sp. B50 isolated from garden soil	Resting cell biotransformation in a mineral medium	-	(Hsiao et al., 2021)
	17α-ethinyl estradiol	0.5 mg/L, 1.4 mg/L	47–48% 35 h	*R. erythropolis*	Modified Mineral Salt Media (MMSM) + 2.5 g/L adipic acid	Metabolite (m/z 331)	(O'Grady et al., 2009)
		0.5 mg/L	10% 22 h	*R. zopfii*		-	
		1.4 mg/L	39% 65 h	*R. equi*	MMSM + 2.5 g/L glucose	-	
		0.5 mg/L	90.4% 48 h	*R. zopfii* ATCC 51349	Small Bioreactor Platform macro-encapsulation method (SBP) in a	-	(Menashe et al., 2020)

Therapeutic class	Compound	Concentration	Biodegradation	Biodegrader	Biodegradation conditions	Metabolites	Reference
					minimal salt medium		
		0.9 mg/L	94.9% 72 h		SBP in a minimal salt medium enriched with 2% lysogeny broth	-	
		2.2 mg/L	91.8% 96 h		SBP in a minimal salt medium enriched with 2% sterilized domestic secondary effluents	-	
		5 mg/L	61% 300 h	*R. equi* ATCC 13557	Minimum mineral salt media (MMSM)	Unidentified metabolite	(Larcher and Yargeau, 2013)
			46% 300 h	*R. erythropolis* ATCC 4277		Unidentified metabolite	
			100% 300 h	*R. rhodocrous* ATCC 13808		-	
			38% 300 h	*R. zopfii* ATCC 51349			
			43% 300 h	*P. aeruginosa* PA01, *P. putida* ATCC 12633, *R. equi* ATCC 13557, *R. erythropolis* ATCC 4277, *R. rhodocrous* ATCC 13808			
			42% 300 h	*B. subtilis* ATCC 6051, *P. putida* ATCC 12633, *R. equi* ATCC 13557, *R. erythropolis*			

Therapeutic class	Compound	Concentration	Biodegradation	Biodegrader	Biodegradation conditions	Metabolites	Reference
				ATCC 4277, *R. rhodocrous* ATCC 13808, *R. zopfii* ATCC 51349			
		30 mg/L	90% 72 h	*R. equi* DSSKP-R-001	Mineral basal medium	-	(Tian et al., 2020)
		100 mg/L	80% 24 h	Individual strains *R. equi* Y 50155, Y 50157 isolated from activated sludge in a wastewater treatment plant	Modified mineral Dominic and Graham's (MDG) culture medium	-	(Yoshimoto et al., 2004)
		100 mg/L	96 24 h	Individual strain *R. equi* Y 50156 isolated from activated sludge in a wastewater treatment plant			
		100 mg/L	100% 24h	*R. zopfii* Y 50158 isolated from activated sludge in a wastewater treatment plant			
	E1+E2	5 mg/L each	≈85% of E2 40 h	*R. equi* ATCC13557	Mineral salts medium	-	(Harthern-Flint et al., 2021)
	E2 + EE2	30 mg/L each	58.7% at 72 h for E2<br><br>97.3% at 97 h for EE2	*R. equi* DSSKP-R-001	Mineral basal medium	-	(Tian et al., 2020)

Therapeutic class	Compound	Concentration	Biodegradation	Biodegrader	Biodegradation conditions	Metabolites	Reference
	Progesterone	500 µg/L	99% 1 h	*Rhodococcus* sp. HYW isolated from activated sludge	Minimum mineral medium	-	(Yu et al., 2018)
		500 µg/L	99% 1.5 h	Activated sludge bioaugmented with *Rhodococcus* sp. HYW	Minimum mineral medium + 35 mg/L glucose	Testosterone; 3β-Hydroxy-5α-pregnan-20-one; 5α-dihydrotestosterone; (8R,9S,13S,14S)-3-hydroxy-13-methyl-1,2,3,6,7,8,9,10,11,12,13,14,15,16-tetradecahydro17H-cyclopenta[a]phenanthren-17-one; estrone; androst-1,4,9(11)-triene-3,17-dione; 3-hydroxy-9,10-seconandrost-1,3,5(10)-triene-9,17-dione; 9,17-dioxo-1,2,3,4,10,19-hexanorandrostan-5-oic acid; 3a-H-4α-[3-propanol]-5α-hydroxy-7aβ-methylhex-ahydro-1-	

Therapeutic class	Compound	Concentration	Biodegradation	Biodegrader	Biodegradation conditions	Metabolites	Reference
						indanone; 2,4,6-trihydroxy-5-methylhexanoic acid	
	Testosterone	100 mg/L	100% 40 h	*Rhodococcus* sp. B50 isolated from garden soil	Resting cell biotransformation in a mineral medium	-	(Hsiao et al., 2021)
		1 g/L	100% 36 h	*R. equi* ATCC 14887	Minimal liquid medium	9a-hydroxyandrost-4-ene-3,17-dione and 9a,17b-dihydroxyandrost-4-en-3-one	(Kim et al., 2007)
Antiplasmodics	Drotaverine	20 mg/L	100% 60 d	*R. rhodochrous* IEGM 608	Free cell (pre-grown on isoquinoline) biodegradation in RS mineral salt medium	3,4-diethoxybenzoic acid (protocatechic acid) derivatives	(Ivshina et al., 2012)
			100% 45 d		Free cell (pre-grown on isoquinoline) biodegradation in RS mineral salt medium + 5 g/L glucose		
			100% 30 d		Immobilized isoquinoline-adapted cell (adsorbed onto pine		

Therapeutic class	Compound	Concentration	Biodegradation	Biodegrader	Biodegradation conditions	Metabolites	Reference
					sawdust) biodegradation in RS mineral salt medium + 5 g/L glucose		
			5-8% 48 h	*R. ruber* IEGM 326	Cyst like dormant cells in RS mineral salt medium	-	(Ivshina et al., 2015)
			46% 48 h		Cyst like dormant cells in RS mineral salt medium RS mineral salt medium + 5 g/L glucose		
Analgesics	Paracetamol	1 mg/L	100% after 24 h	*R. erythropolis* BIOMIG-P19	MSM	p-aminophenol	(Akay and Tezel, 2020)
		10 mg/L	100% after 12 h				
		100, 500 mg/L	100% after 6 h				
		20 mg/L	100% after 5 d	*R. ruber* IEGM 77	RS minimum mineral medium, paracetamol was used in the form of pills	-	(Ivshina et al., 2006)
		500 mg/L	77% after 20 d		K minimum mineral medium		

Therapeutic class	Compound	Concentration	Biodegradation	Biodegrader	Biodegradation conditions	Metabolites	Reference
			86% after 20 d		RS minimum mineral medium	p-aminophenol, pyrocatechol, hydroquinone	
NSAIDs	Diclofenac	50 mg/L	≈50% after 60 d	*R. ruber* IEGM 231, IEGM 346	RS minimum mineral medium + 0.5% glucose + preliminary cell adaptation in the presence of 5 mg/L diclofenac	16 metabolites, C-N bond cleavage, aromatic ring opening. Terminal products: fumarial acetoacetic acid and its derivatives (fumaric and acetoacetic acids)	(Ivshina et al., 2019; Tyumina et al., 2019)
		50 µg/L	100% after 6 d	*R. ruber* IEGM 346	RS minimum mineral medium + 0.5% glucose + preliminary cell adaptation in the presence of 5 µg/L diclofenac		(Ivshina et al., 2019)
	Ibuprofen	100 mg/L	100% after 144 h	*R. cerastii* IEGM 1278	RS mineral salt medium + 0.1 vol. % n-hexadecane	9-hydroxy ibuprofen; 6,9-dihydroxy ibuprofen; 6-hydroxy ibuprofen; decarboxylated derivative of 9-hydroxy ibuprofen; decarboxylated derivative of 6,9-dihydroxy ibuprofen; decarboxylated	(Ivshina et al., 2021)
		100 µg/L	100% after 30 h				

Therapeutic class	Compound	Concentration	Biodegradation	Biodegrader	Biodegradation conditions	Metabolites	Reference
						derivative of 6-hydroxy ibuprofen	
	Ketoprofen	100 mg/L	38% after 14 d	*R. erythropolis* IEGM 746	RS mineral salt medium + 0.1 vol. % n-hexadecane	-	(Bazhutin et al., 2022)
	Acetylsalicylic acid	250 mg/L	100% after 11 d	*R. jostii* IEGM 60	RS mineral salt medium	Salicylic acid	(Khrenkov et al., 2020)
		250 mg/L	100% after 9 d		RS mineral salt medium + polyvinylpyrrolidone		
		250 mg/L	100% after 6 d		RS mineral salt medium, paracetamol was used in the form of pills		
Antiepileptics	Carbamazepine	9.5 ppm	20% 28 d	*R. rhodochrous* ATC 13808	Minimum mineral salt media (MMSM) + 3 g/L glucose	-	(Gauthier et al., 2010)
Lipid-lowering drugs	Clofibric acid	0.1 g/L	~100% 20 d	*R. rhodochrous* ATCC 13808	Minimal mineral salts media + 0.1 g/L yeast extract, 2.5 g/L glucose	Clofibrate	(Evangelista et al., 2008)

- Not identified.

References

Akay, C., and Tezel, U. (2020). Biotransformation of Acetaminophen by intact cells and crude enzymes of bacteria: A comparative study and modelling. *Science of The Total Environment* 703, 134990. doi: 10.1016/j.scitotenv.2019.134990.

Bazhutin, G. A., Polygalov, M. A., Tyumina, E. A., Tyan, S. M., and Ivshina, I. B. (2022). Cometabolic bioconversion of ketoprofen by *Rhodococcus erythropolis* IEGM 746. in *Science and Global Challenges of the 21st Century - Science and Technology. Perm Forum 2021*, eds. A. Rocha and E. Isaeva (Cham: Springer), 404–410. doi: 10.1007/978-3-030-89477-1_40.

Bouju, H., Ricken, B., Beffa, T., Corvini, P. F. X., and Kolvenbach, B. A. (2012). Isolation of bacterial strains capable of sulfamethoxazole mineralization from an acclimated membrane bioreactor. *Applied and Environmental Microbiology* 78, 277–279. doi: 10.1128/AEM.05888-11.

Evangelista, S., Yargeau, V., and Cooper, D. G. (2008). The recalcitrance of clofibric acid to microbial degradation. *WIT Transactions on Ecology and the Environment* 111, 273–278. doi: 10.2495/WP080271.

Gauthier, H., Yargeau, V., and Cooper, D. G. (2010). Biodegradation of pharmaceuticals by *Rhodococcus rhodochrous* and *Aspergillus niger* by co-metabolism. *Science of the Total Environment* 408, 1701–1706. doi: 10.1016/j.scitotenv.2009.12.012.

Harthern-Flint, S. L., Dolfing, J., Mrozik, W., Meynet, P., Eland, L. E., Sim, M., et al. (2021). Experimental and genomic evaluation of the oestrogen degrading bacterium *Rhodococcus equi* ATCC13557. *Frontiers in Microbiology* 12, 670928. doi: 10.3389/FMICB.2021.670928.

Hsiao, T. H., Chen, Y. L., Meng, M., Chuang, M. R., Horinouchi, M., Hayashi, T., et al. (2021). Mechanistic and phylogenetic insights into actinobacteria-mediated oestrogen biodegradation in urban estuarine sediments. *Microbial Biotechnology* 14, 1212–1227. doi: 10.1111/1751-7915.13798.

Ivshina, I. B., Mukhutdinova, A. N., Tyumina, H. A., Vikhareva, H. V., Suzina, N. E., El'-Registan, G. I., et al. (2015). Drotaverine hydrochloride degradation using cyst-like dormant cells of *Rhodococcus ruber*. *Current Microbiology* 70, 307–314. doi: 10.1007/s00284-014-0718-1.

Ivshina, I. B., Rychkova, M. I., Vikhareva, E. V., Chekryshkina, L. A., and Mishenina, I. I. (2006). Catalysis of the biodegradation of unusable medicines by alkanotrophic rhodococci. *Applied Biochemistry and Microbiology* 42, 392–395. doi: 10.1134/S0003683806040090.

Ivshina, I. B., Tyumina, E. A., Bazhutin, G. A., and Vikhareva, E. V. (2021). Response of *Rhodococcus cerastii* IEGM 1278 to toxic effects of ibuprofen. *PLoS ONE* 16, e0260032. doi: 10.1371/JOURNAL.PONE.0260032.

Ivshina, I. B., Tyumina, E. A., Kuzmina, M. V., and Vikhareva, E. V. (2019). Features of diclofenac biodegradation by *Rhodococcus ruber* IEGM 346. *Scientific Reports* 9, 9159. doi: 10.1038/s41598-019-45732-9.

Ivshina, I. B., Vikhareva, E. V., Richkova, M. I., Mukhutdinova, A. N., and Karpenko, J. N. (2012). Biodegradation of drotaverine hydrochloride by free and immobilized cells of *Rhodococcus rhodochrous* IEGM 608. *World Journal of Microbiology and Biotechnology* 28, 2997–3006. doi: 10.1007/s11274-012-1110-6.

Khrenkov, A. N., Vikhareva, E. V., Tumilovich, E. Yu., Karpenko, Yu. N., Selyaninov, A. A., and Tyumina, E. A. (2020). Chromatographic analysis of acetylsalicylic acid in *Rhodococcus* cultural fluids. *Moscow University Chemistry Bulletin* 75, 309–314. doi: 10.3103/S0027131420050053.

Kim, Y. U., Han, J., Sang, S. L., Shimizu, K., Tsutsumi, Y., and Kondo, R. (2007). Steroid 9α-hydroxylation during testosterone degradation by resting *Rhodococcus equi* cells. *Arch Pharm (Weinheim)* 340, 209–214. doi: 10.1002/ardp.200600175.

Kurisu, F., Ogura, M., Saitoh, S., Yamazoe, A., and Yagi, O. (2010). Degradation of natural estrogen and identification of the metabolites produced by soil isolates of *Rhodococcus* sp. and *Sphingomonas* sp. *Journal of Bioscience and Bioengineering* 109, 576–582. doi: 10.1016/j.jbiosc.2009.11.006.

Larcher, S., and Yargeau, V. (2011). Biodegradation of sulfamethoxazole by individual and mixed bacteria. *Applied Microbiology and Biotechnology* 91, 211–218. doi: 10.1007/s00253-011-3257-8.

Larcher, S., and Yargeau, V. (2012). The effect of ozone on the biodegradation of 17α-ethinylestradiol and sulfamethoxazole by mixed bacterial cultures. *Applied Microbiology and Biotechnology* 97, 2201–2210. doi: 10.1007/S00253-012-4054-8.

Larcher, S., and Yargeau, V. (2013). Biodegradation of 17α-ethinylestradiol by heterotrophic bacteria. *Environmental Pollution* 173, 17–22. doi: 10.1016/j.envpol.2012.10.028.

Lee, D. G., and Chu, K.-H. (2013). Effects of growth substrate on triclosan biodegradation potential of oxygenase-expressing bacteria. *Chemosphere* 93, 1904–1911. doi: 10.1016/j.chemosphere.2013.06.069.

Li, C., Sun, Y., Sun, G., Zang, H., Sun, S., Zhao, X., et al. (2022). An amidase and a novel phenol hydroxylase catalyze the degradation of the antibacterial agent triclocarban by *Rhodococcus rhodochrous*. *Journal of Hazardous Materials* 430, 128444. doi: 10.1016/J.JHAZMAT.2022.128444.

Li, Z., Dai, R., Yang, B., Chen, M., Wang, X., and Wang, Z. (2021). An electrochemical membrane biofilm reactor for removing sulfonamides from wastewater and suppressing antibiotic resistance development: Performance and mechanisms. *Journal of Hazardous Materials* 404, 124198. doi: 10.1016/j.jhazmat.2020.124198.

Liu, J., Liu, J., Xu, D., Ling, W., Li, S., and Chen, M. (2016). Isolation, immobilization, and degradation performance of the 17β-estradiol-degrading bacterium *Rhodococcus* sp. JX-2. *Water, Air, & Soil Pollution* 227, 422. doi: 10.1007/s11270-016-3122-6.

Maia, A. S., Tiritan, M. E., and Castro, P. M. L. (2018). Enantioselective degradation of ofloxacin and levofloxacin by the bacterial strains *Labrys portucalensis* F11 and *Rhodococcus* sp. FP1. *Ecotoxicology and Environmental Safety* 155, 144–151. doi: 10.1016/j.ecoenv.2018.02.067.

Menashe, O., Raizner, Y., Kuc, M. E., Cohen-Yaniv, V., Kaplan, A., Mamane, H., et al. (2020). Biodegradation of the endocrine-disrupting chemical 17α-ethynylestradiol (EE2) by *Rhodococcus zopfii* and *Pseudomonas putida* encapsulated in small bioreactor platform (SBP) capsules. *Applied Sciences* 10, 336. doi: 10.3390/APP10010336.

O'Grady, D., Evangelista, S., and Yargeau, V. (2009). Removal of aqueous 17α-ethinylestradiol by *Rhodococcus* species. *Environmental Engineering Science* 26, 1393–1400. doi: 10.1089/ees.2008.0272.

Tian, K., Meng, F., Meng, Q., Gao, Y., Zhang, L., Wang, L., et al. (2020). The analysis of estrogen-degrading and functional metabolism genes in *Rhodococcus equi* DSSKP-R-001. *International Journal of Genomics* 2020, 9369182. doi: 10.1155/2020/9369182.

Tyumina, E. A., Bazhutin, G. A., Vikhareva, E. V., Selyaninov, A. A., and Ivshina, I. B. (2019). Diclofenac as a factor in the change of *Rhodococcus* metabolism. *IOP Conference Series: Materials Science and Engineering* 487, 012027. doi: 10.1088/1757-899X/487/1/012027.

Wang, Y., Shao, H., Zhu, S., Tian, K., Qiu, Q., and Huo, H. (2019). Degradation of 17β-estradiol and products by a mixed culture of *Rhodococcus equi* DSSKP-R-001 and *Comamonas testosteroni* QYY20150409. *Biotechnology and Biotechnological Equipment* 33, 268–277. doi: 10.1080/13102818.2019.1568913.

Yeom, J. R., Yoon, S. U., and Kim, C. G. (2017). Quantification of residual antibiotics in cow manure being spread over agricultural land and assessment of their behavioral effects on antibiotic resistant bacteria. *Chemosphere* 182, 771–780. doi: 10.1016/j.chemosphere.2017.05.084.

Yoshimoto, T., Nagai, F., Fujimoto, J., Watanabe, K., Mizukoshi, H., Makino, T., et al. (2004). Degradation of estrogens by *Rhodococcus zopfii* and *Rhodococcus equi* isolates. *Applied and Environmental Microbiology* 70, 5283–5289. doi: 10.1128/AEM.70.9.5283.

Yu, Q., Geng, J., Huo, H., Xu, K., Huang, H., Hu, H., et al. (2018). Bioaugmentated activated sludge degradation of progesterone: Kinetics and mechanism. *Chemical Engineering Journal* 352, 214–224. doi: 10.1016/j.cej.2018.06.159.

Zhang, J., Chen, R., Du, C., Dong, S., and Sun, J. (2021). Effects of continuous sulfamonomethoxine shock on the power generation performance and microbial community structure of MFCs under seasonal temperature variation. *Biochemical Engineering Journal* 167, 107909. doi: 10.1016/j.bej.2020.107909.
