# Supplementary figures and images for: Rhodococcus strains as a good biotool for neutralizing pharmaceutical pollutants and obtaining therapeutically valuable products: Through the past into the future
Source: Front Microbiol. 2022 Sep 29;13:967127. doi: 10.3389/fmicb.2022.967127 (PMC9557007; doi:10.3389/fmicb.2022.967127)

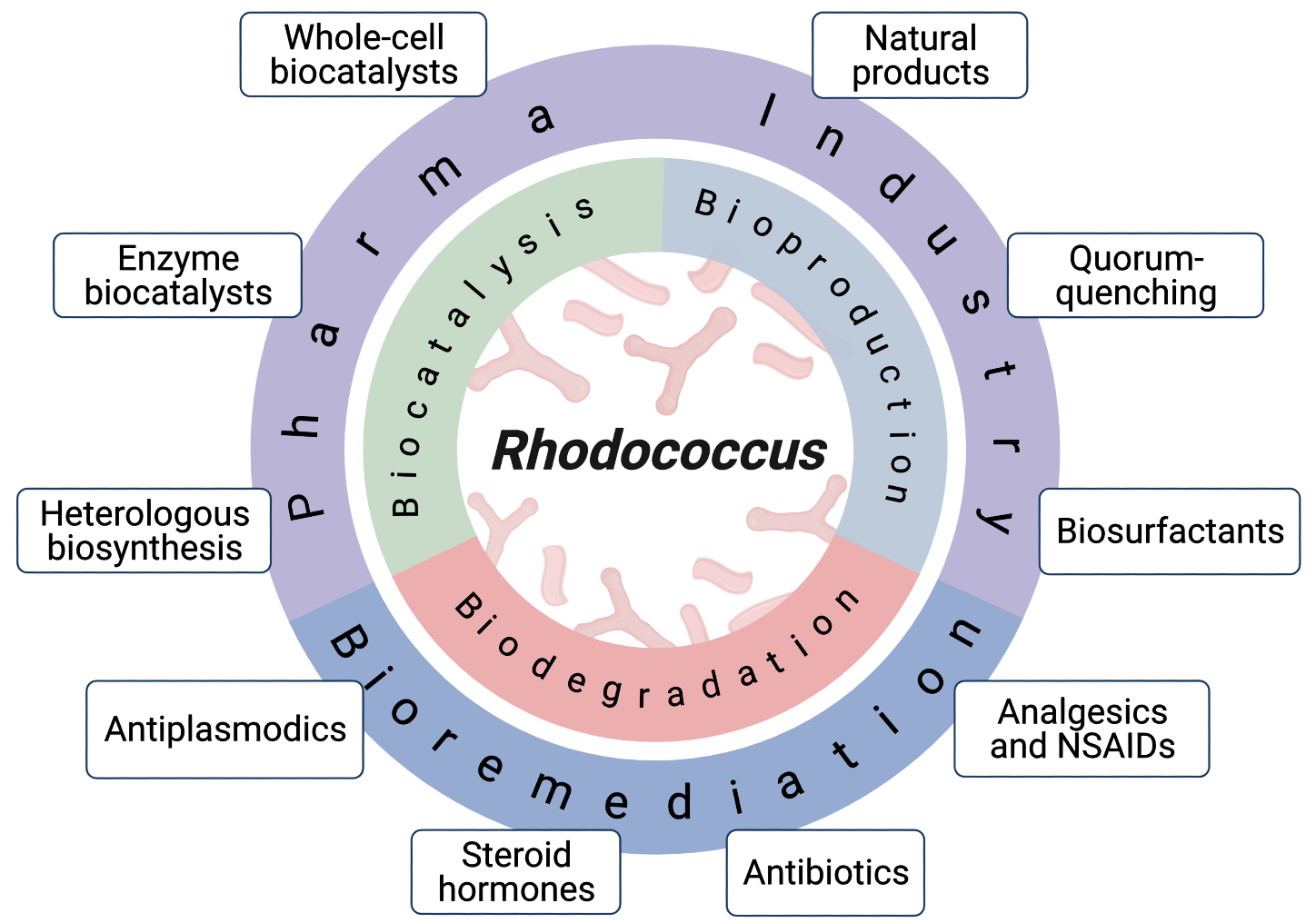

Supplement: Supplementary file 2 [file Image_1.TIFF]
