# Supplementary material for: Rhodococcus strains as a good biotool for neutralizing pharmaceutical pollutants and obtaining therapeutically valuable products: Through the past into the future
Source: Front Microbiol. 2022 Sep 29;13:967127. doi: 10.3389/fmicb.2022.967127 (PMC9557007; doi:10.3389/fmicb.2022.967127)
Supplement: Supplementary file 3 [file Image_2.PDF]

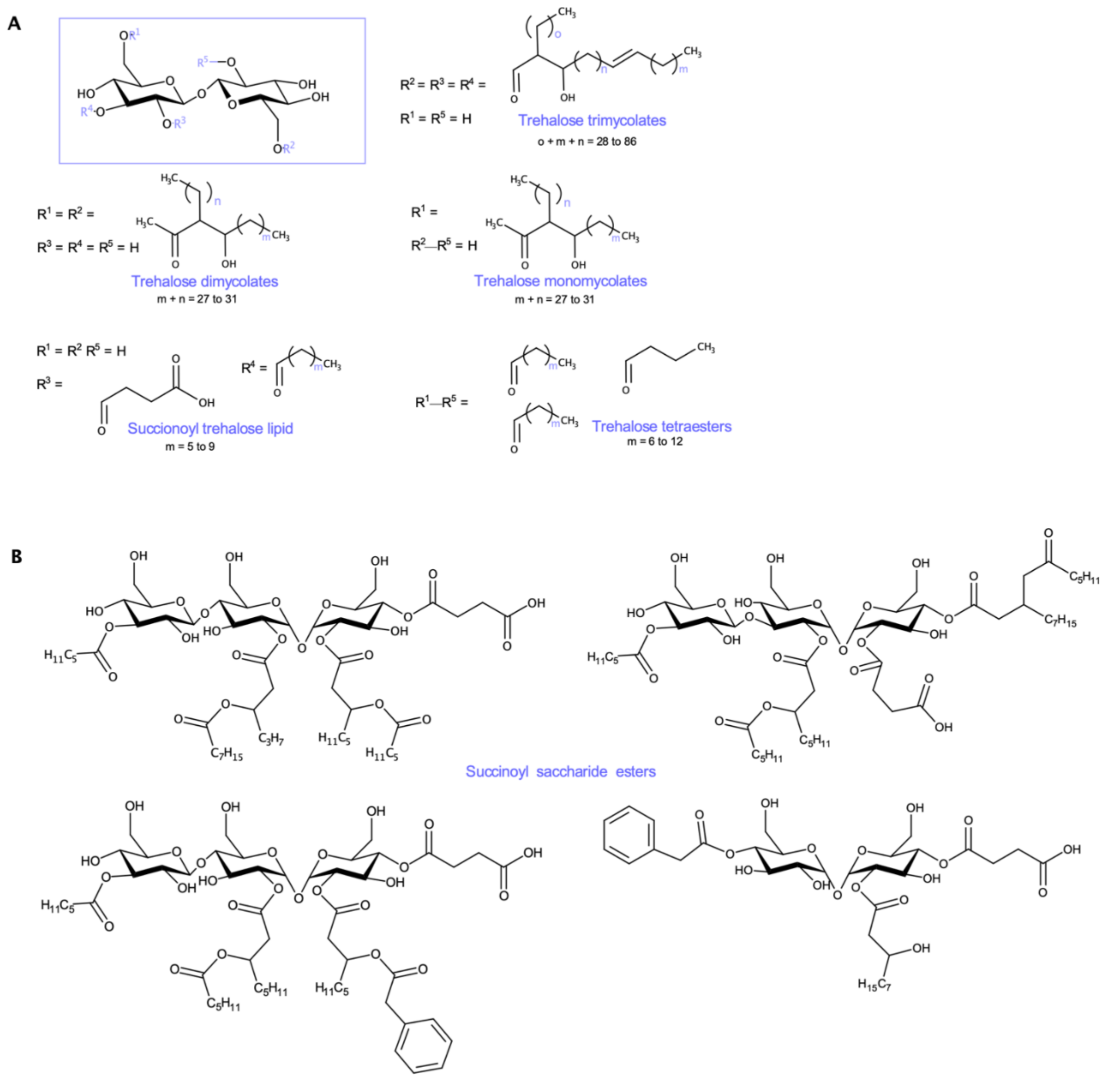

Supplementary Figure 1. Chemical structures of mostly known trehalolipids from *Rhodococcus* (A) and succinoyl saccharide esters isolated from the deep-sea *Rhodococcus* sp. BS-15 (B) (Kuyukina and Ivshina, 2019; Esposito et al., 2021).

References

Esposito, F. P., Giugliano, R., Sala, G. della, Vitale, G. A., Buonocore, C., Ausuri, J., et al. (2021). Combining OSMAC approach and untargeted metabolomics for the identification of new glycolipids with potent antiviral activity produced by a marine *Rhodococcus*. *International Journal of Molecular Sciences* 22, 9055. doi: 10.3390/IJMS22169055.

Kuyukina, M. S., and Ivshina, I. B. (2019). "Production of trehalolipid biosurfactants by *Rhodococcus*," in *Biology of Rhodococcus*, ed. H. M. Alvarez (Cham: Springer), 271–298. doi: 10.1007/978-3-030-11461-9_10.
